# Tripartite Motif 22 (TRIM22) protein restricts herpes simplex virus 1 by epigenetic silencing of viral immediate-early genes

**DOI:** 10.1371/journal.ppat.1009281

**Published:** 2021-02-01

**Authors:** Tejaswini S. Reddi, Philipp E. Merkl, So-Yon Lim, Norman L. Letvin, David M. Knipe

**Affiliations:** 1 Department of Microbiology, Blavatnik Institute, Harvard Medical School, Boston, Massachusetts, United States of America; 2 Center for Virology and Vaccine Research, Beth Israel Deaconess Medical Center, Harvard Medical School, Boston, Massachusetts, United States of America; State University of New York Upstate Medical University, UNITED STATES

## Abstract

Intrinsic resistance is a crucial line of defense against virus infections, and members of the Tripartite Ring Interaction Motif (TRIM) family of proteins are major players in this system, such as cytoplasmic TRIM5α or nuclear promyelocytic leukemia (PML/TRIM19) protein. Previous reports on the antiviral function of another TRIM protein, TRIM22, emphasized its innate immune role as a Type I and Type II interferon-stimulated gene against RNA viruses. This study shows that TRIM22 has an additional intrinsic role against DNA viruses. Here, we report that TRIM22 is a novel restriction factor of HSV-1 and limits ICP0-null virus replication by increasing histone occupancy and heterochromatin, thereby reducing immediate-early viral gene expression. The corresponding wild-type equivalent of the virus evades the TRIM22-specific restriction by a mechanism independent of ICP0-mediated degradation. We also demonstrate that TRIM22 inhibits other DNA viruses, including representative members of the β- and γ- herpesviruses. Allelic variants in *TRIM22* showed different degrees of anti-herpesviral activity; thus, *TRIM22* genetic variability may contribute to the varying susceptibility to HSV-1 infection in humans. Collectively, these results argue that TRIM22 is a novel restriction factor and expand the list of restriction factors functioning in the infected cell nucleus to counter DNA virus infection.

## Author summary

The host immune response to herpesviruses includes intrinsic immunity, which is a constitutively active line of defense. Members of the Tripartite Motif (TRIM) superfamily of proteins, such as cytoplasmic TRIM5α and nuclear TRIM19, are examples of such restriction factors against the prototypical α-herpesvirus, herpes simplex virus-1 (HSV-1). Previous reports on the antiviral function of the protein encoded by *TRIM22*, a gene closely related to the *TRIM5* gene, emphasized its antiretroviral role. We show that *TRIM22* has an additional role as a restriction factor against herpesviruses. We found that TRIM22 inhibits a mutant form of HSV-1, by promoting chromatin compaction of the viral DNA encoding immediate-early viral genes–this consequently inhibits viral replication and reduces virus yields. Unlike other restriction factors that are degraded by the viral infected cell polypeptide 0 (ICP0), TRIM22 is not degraded by ICP0. We also show that TRIM22 inhibits representative members of the β-herpesvirus (cytomegalovirus) and γ- herpesviruses (Epstein-Barr virus). In addition, different *TRIM22* genetic variants show differing levels of HSV-1 inhibition. Together, these results argue for the importance of the *TRIM22* gene as a restriction factor against herpesviruses, and offer a novel avenue for further investigation on the role of *TRIM* genes in host genetic variation in herpesviral susceptibility.

## Introduction

The cellular intrinsic resistance system consists of constitutively expressed, germline-encoded restriction factors that provide an immediate anti-viral response in host cells [1]. This system includes Tripartite Ring Interaction Motif (TRIM) proteins, which have important roles in viral inhibition in addition to being involved in diverse cellular processes [2–6]. For the nuclear-replicating DNA herpesvirus family of viruses, nuclear domain 10 (ND10) bodies are well-established nuclear sub-domains that confer resistance to the ubiquitous herpesviruses [7]. They consist of proteins including TRIM19 or promyelocytic leukemia protein (PML), Sp100, death domain associated protein (hDaxx), and alpha-thalassemia/mental retardation syndrome X-linked protein (ATRX) [8]. PML, Sp100, hDaxx and ATRX depletion increase the infectivity of a defective herpes simplex virus 1 (HSV-1) virus deficient in the viral transactivator infected cell polypeptide 0 (ICP0-null HSV-1) [9–11]. hDaxx has been shown to repress human cytomegalovirus (HCMV) and a replication-defective version of HCMV [12]. Both hDaxx and ATRX have been shown to be important in chromatin modification and viral gene repression [13,14].

To counteract the restriction exerted by the various known intrinsic immune factors, the herpesviruses encode a number of proteins that allow for virus propagation, even in relatively hostile conditions implemented by the host interferon (IFN) responses [15]. This includes HSV-1 ICP0, which antagonizes nuclear ND10 bodies and gamma-interferon-inducible protein 16 (IFI16) and other components of the intrinsic immune system [15–19]. ICP0 also sequesters interferon regulatory factor 3 (IRF3) from the *IFNB* gene promoter, thus preventing the expression of Type I IFN [15,20,21]. In addition, ICP0 counters host chromatin silencing mechanisms, allowing for the de-repression of early (E) genes [22,23]. As a result, ICP0-null viruses demonstrate a 10-1000-fold replication defect relative to the wild-type viruses in primary cells [24,25]. Because depletion of known restriction factors of HSV-1 such as PML and IFI16 rescue only a portion of the defect in ICP0-null virus replication [10], we hypothesized that there were other factors that could contribute to this restriction.

One such candidate was the human TRIM22 protein, which is the paralog of the prototypical TRIM protein of the rhesus macaque TRIM5α (rhTRIM5α) [26]. rhTRIM5α was reported to inhibit lentiviruses, and we reported previously that rhTRIM5α also inhibits HSV-1 [27]. Sequence analyses of rh*TRIM5* and human *TRIM22* genes from 27 primate genomes demonstrated that both the *TRIM5* and *TRIM22* genes underwent positive selection such that either the *TRIM5* or *TRIM22* gene was selectively modified within a given species, arguing for selective evolutionary pressure exerted by species-specific pathogens [28]. This positive selection resulted in *TRIM22* variation at the level of single nucleotide polymorphisms (SNPs), which have been shown to be associated with functional anti-viral significance [29].

The characterized variation in the human *TRIM22* gene that alter its antiviral function include, but are not limited to, the following SNPs: rs10838543 T/C, rs7935564 A/G and rs1063303 C/G [30]. For example, HIV-1 replication was higher in peripheral blood mononuclear cells (PBMCs) from human donors homozygous at SNPs rs7935564 G/G and rs1063303 G/G [31]. In contrast, the *TRIM22* SNP rs1063303 C/C decreased TRIM22’s antiviral function against HIV-1 [32]. Furthermore, the *TRIM22* SNP rs10838543 C/C genotype in the Han-Chinese population is associated with chronic hepatitis B virus (HBV) infection [33]. Collectively, these studies provide evidence for the importance of genetic variation in the *TRIM22* gene in the anti-viral response.

Furthermore, TRIM22 has been characterized as an interferon (IFN)-inducible protein with antiviral activity against a range of viruses, particularly RNA viruses [34–37] and the hepatitis B virus (HBV), a DNA virus [38]. In addition, TRIM22 has an additional function as an interferon-stimulated gene (ISG) and its expression has also been correlated with the induction of an anti-viral state upon establishment of latency in members of the γ-herpesvirus family such as Kaposi’s sarcoma herpes virus (KSHV) [39,40] and the Epstein-Barr virus (EBV) [40,41]. Furthermore, TRIM22 was previously reported to be upregulated in the first 24h of infection in primary human foreskin fibroblasts (HFFs) with human cytomegalovirus (HCMV), a β-herpesvirus [42]. However, the role of TRIM22 as an intrinsic immune factor in α-herpesviruses, such as HSV-1 infection, has not been investigated.

Here, we report that TRIM22 limits ICP0-null virus replication by modulating histone occupancy and heterochromatin and reducing immediate-early (IE) viral gene expression. The ICP0-rescued virus partially evades the TRIM22-specific restriction by a mechanism independent of ICP0-mediated degradation. Our study shows that depletion of TRIM22 rescues part of the replication defect of the ICP0-null virus. In addition, we also find that the restrictive effect of TRIM22 is not limited to HSV-1, but is also seen with other herpesviruses that replicate in the nucleus, such as HCMV and EBV. We also report that the haplotypic variation in the *TRIM22* gene [43], affects the anti-herpesviral activity of TRIM22 in cell culture studies. Consequently, we speculate that the genetic variation observed at the *TRIM22* gene locus is due to evolutionary pressure exerted by herpesviruses, in addition to the previously known retroviruses. These results establish that TRIM22 is a novel component of the intrinsic immune response to the herpesviruses.

## Results

### Depletion of TRIM22 increases ICP0-null HSV replication in normal human foreskin fibroblasts

**Fig 1:**
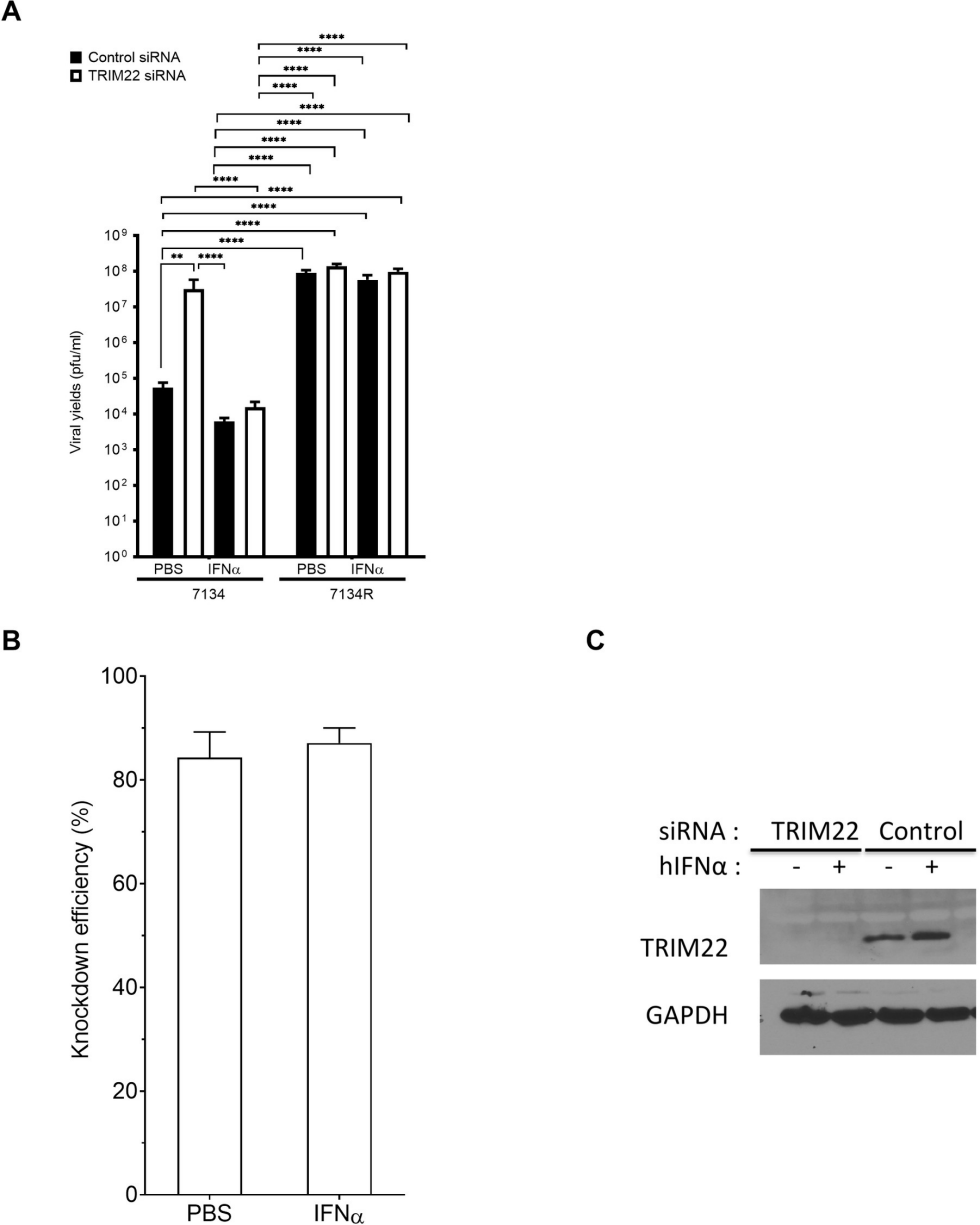
TRIM22 depletion increases HSV-1 viral yields and gene expression. HFFs were transfected with siRNA pools specific for TRIM22 or non-targeting siRNAs. Transfected HFFs were treated with PBS or hIFNα-2a at 1000U/ml for 24 h. These HFFs were then infected with HSV-1 ICP0-null (7134) or the rescued virus (7134R) at an MOI of 5 for 24 h. (A) Virus yields were measured at 24 hpi using plaque assays on U2OS cells (n = 3). Error bars represent standard errors of the means (*P*<0.05*, *P<0*.005**, *P<0*.0002***,*P<0*.0001**** Two-way ANOVA, multiple comparisons with Tukey’s corrections). (B) TRIM22 transcript levels were measured by qRT-PCR with primers specific for *TRIM22* transcripts and 18S rRNA (n = 3). (C) Protein levels were determined by immunoblotting for TRIM22 and GAPDH at time of infection.

To determine the role of TRIM22 as an interferon-stimulated gene (ISG) in HSV-1 replication, we measured virus yields of the HSV-1 7134 ICP0-null virus or the corresponding 7134R rescued virus in TRIM22-depleted human foreskin fibroblast (HFF) cells with or without IFNα pre-treatment. We transfected primary human foreskin fibroblasts (HFFs) twice with pooled siRNAs specific for *TRIM22* to deplete *TRIM22* mRNA transcripts or non-targeting siRNAs as a control. To upregulate *TRIM22* expression, we treated the transfected cells with either PBS or IFNα (1000U/ml, 24 h). We then infected these cells with the HSV-1 7134 ICP0-null or 7134R ICP0-restored viruses at a multiplicity of infection (MOI) of 5 and measured virus yields at 24 h post infection (hpi). We observed an increase in 7134 virus yields in TRIM22-depleted HFFs relative to control HFFs under conditions of PBS pre-treatment (Fig 1A). IFNα pre-treatment reduced 7134 virus yields by approximately 10-fold relative to PBS pre-treatment in HFFs, and TRIM22 depletion partially reversed IFNα-mediated inhibition of 7134 (Fig 1A). Upon comparison of TRIM22 transcripts in control-depleted and TRIM22-depleted fibroblasts, the efficiency of knockdown was approximately 84% in the PBS pre-treated cells and 87% in the IFNα pre-treated cells (Fig 1B). The levels of TRIM22 protein were not detectable by western blotting in TRIM22-depleted HFFs; IFNα pre-treatment increased TRIM22 protein levels in control-depleted HFFs (Fig 1C). These results indicated that TRIM22 1) under basal expression levels inhibits the replication of an ICP0-null virus and 2) under IFNα-induced levels exerts part of the enhanced antiviral effect of Type 1 IFN on ICP0-null virus. In addition, these results argued that an MOI of 5 is insufficient for the HSV-1 7134 ICP0-null virus to overcome the TRIM22-mediated inhibition of viral replication.

### Over-expression of TRIM22 reduces ICP0-null and ICP0-rescued virus replication

**Fig 2:**
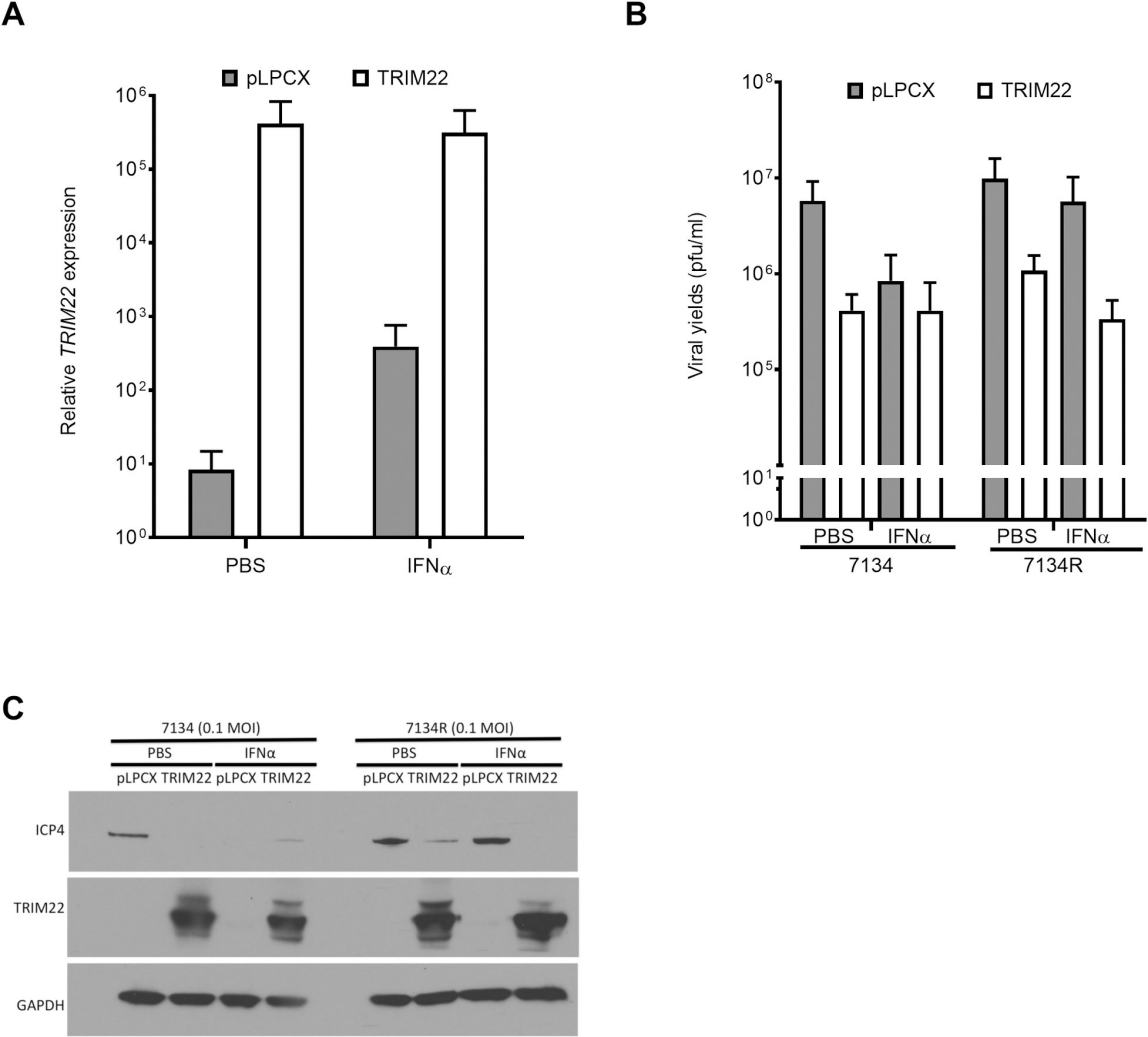
Overexpression of TRIM22 reduces HSV-1 replication. HeLa cells transfected with an empty vector control (pLPCX) or a vector with a TRIM22 insert (TRIM22) were treated with PBS or hIFNα-2a at 1000U/ml for 24 h. Transfected cells were infected with ICP0-null (7134) or rescued virus (7134R) at an MOI of 0.1. (A) Total cell-associated RNA was harvested at time of infection and prepared for qRT-PCR. *TRIM22* transcripts were normalized to 18S rRNA. (B) Virus yields at 48 hpi were determined with plaque assays on U2OS cells. (C) Representative western blot showing total cell lysates probed for ICP4, TRIM22, and GAPDH protein levels at 24 hpi (n = 4). (*P*<0.05*, *P<0*.005**, *P<0*.0002***,*P<0*.0001**** Two-way ANOVA, multiple comparisons with Tukey’s corrections).

To confirm that TRIM22 is specifically restricting HSV-1 replication and to test whether TRIM22 was sufficient for the inhibition of the 7134 virus, we measured virus yields in HeLa cells transfected with either a plasmid encoding a prevalent form of *TRIM22* (TRIM22 haplotype #5) [43] or a control plasmid (pLPCX). We chose HeLa cells because they express *TRIM22* transcripts at relatively low levels (S1 Fig), and HeLa cells were also used as cell lines of choice in prior publications of TRIM22 [34,35,44,45]. We observed that IFNα pre-treatment increased *TRIM22* transcripts by approximately 10-fold in pLPCX-transfected HeLa cells, and transfection with *TRIM22*-encoding plasmid increased *TRIM22* transcripts by approximately 10^5^-fold relative to transfection with the empty vector in PBS pre-treated cells, and by approximately 10^3^-fold in IFNα pre-treated cells (Fig 2A). TRIM22 over-expression in HeLa cells reduced both 7134 and 7134R virus yields by approximately 10-fold at an MOI of 0.1 pfu/cell relative to pLPCX transfection (Fig 2B). The magnitude of this difference was negligible at a high MOI of 5 in HeLa cells, despite high levels of expression of the *TRIM22* transcripts upon transfection with the *TRIM22*-encoding plasmid (S2A and S2B Fig). This MOI-dependent effect was consistent with a previous study using exogenous expression systems of the rhesus paralog, rhTRIM5α, where the rhTRIM5α -mediated inhibition of HSV-1 is overcome at higher MOI [27]. The effect of TRIM22 over-expression on 7134R virus yields in conditions of IFNα pre-treatment were comparable to the corresponding virus yields in the control conditions of PBS pre-treatment at an MOI of 0.1 pfu/cell (Fig 2B). Furthermore, exogenous expression of TRIM22 protein reduced IE (ICP4) protein expression (Fig 2C). We confirmed that TRIM22 was capable of reducing IE and E gene expression in the first round of viral replication at this low MOI by measuring viral transcript levels at 4 hpi and 8 hpi (S3 Fig). We found that exogenous expression of TRIM22 reduced IE *ICP27* transcript levels by 4 hpi in 7134R virus-infected cells, and by 8 hpi in 7134 virus-infected cells, before viral spread (S3A and S3B Fig). Therefore, these results agreed with the RNA interference studies and demonstrated that TRIM22 is sufficient for the inhibition of the ICP0-null virus by reducing viral IE gene expression.

### Depletion of TRIM22 enhances HSV-1 IE viral gene expression

**Fig 3:**
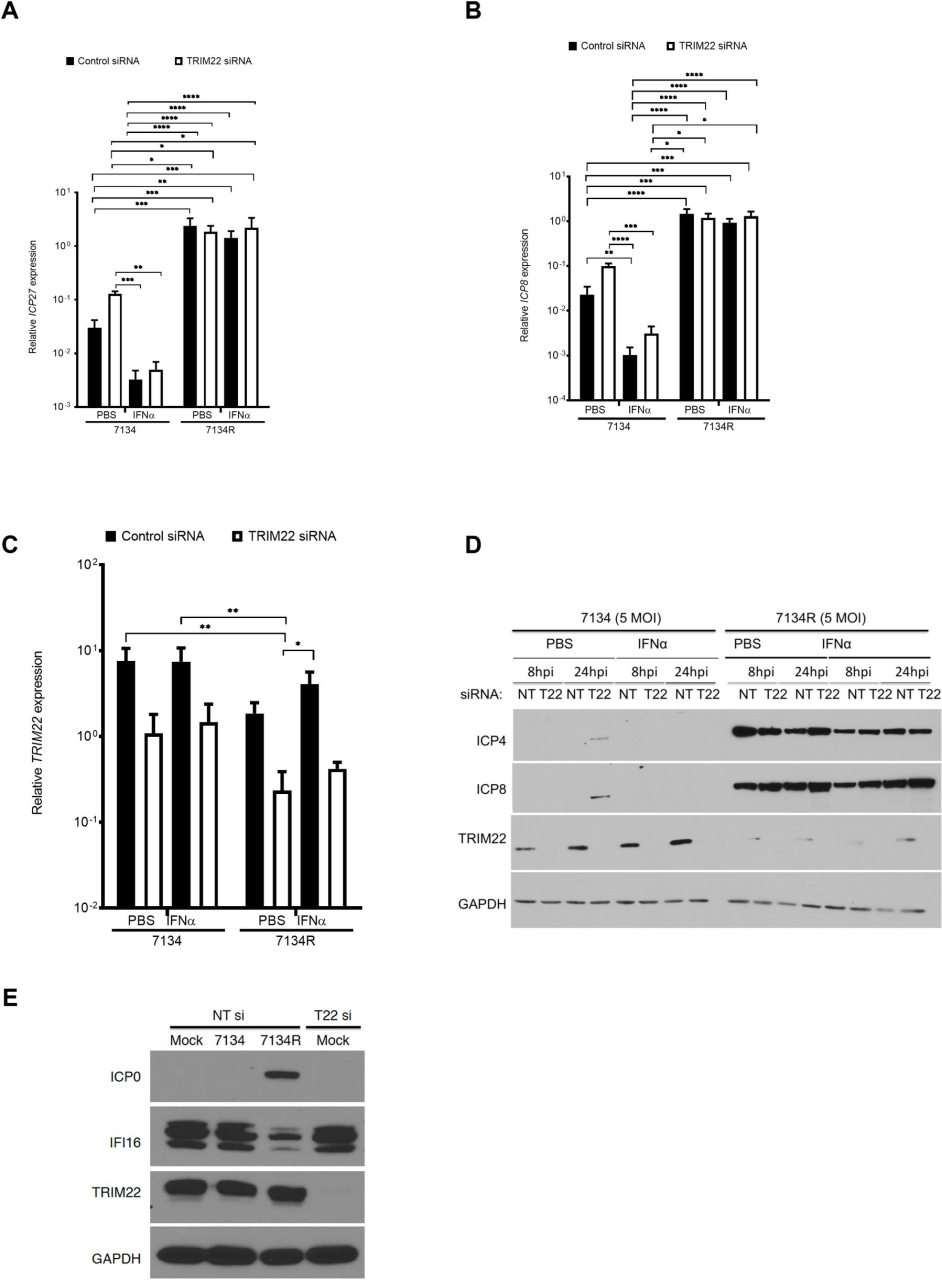
TRIM22 depletion increases viral IE, E and L gene transcripts. Control siRNA or TRIM22 siRNA transfected HFFs treated with PBS or hIFNα-2a at 1000U/ml for 24 h were infected with HSV-1 ICP0-null (7134) or a rescued virus (7134R) at an MOI of 5. (A-C) Total cell-associated RNA was harvested at 8hpi and prepared for qRT-PCR. *ICP27* transcripts (A), *ICP8* transcripts (B), and *TRIM22* transcripts (C) were measured and normalized to *18S* rRNA (n = 3). (D) Whole cell lysates were collected at 24 hpi and immunoblotted for ICP4, ICP8, TRIM22, and GAPDH protein levels. (E) Control siRNA or TRIM22 siRNA transfected HFFs were infected with HSV-1 ICP0-null (7134) or a rescued virus (7134R) or mock-infected at an MOI of 5. Whole cell lysates were collected at 8 hpi and immunoblotted for ICP0, TRIM22, IFI16, and GAPDH protein levels. (*P<0*.05*, *P<0*.005**, *P<0*.0002***,*P<0*.0001**** Two-way ANOVA, multiple comparisons with Tukey’s corrections).

After HSV-1 enters the nucleus, the replication cycle initiates with transcription of immediate-early (IE) genes, followed by early (E) gene transcription [46]. To characterize the TRIM22-mediated block in HSV-1 replication, we measured the transcript levels of IE (*ICP27*) and E (*ICP8*) viral genes in TRIM22-depleted fibroblasts relative to control-depleted fibroblasts in 7134 and 7134R virus infected cells. TRIM22 depletion increased the expression of *ICP27* and *ICP8* transcript levels by approximately 3-fold upon infection with the 7134 virus in control fibroblasts at 8 hpi (Fig 3A and 3B). The TRIM22-mediated effect on *ICP27* and *ICP8* levels under conditions of IFNα pre-treatment was also not significant, but followed the same trend observed in conditions of PBS pre-treatment (Fig 3A and 3B). TRIM22 depletion consistently reduced *TRIM22* transcripts relative to non-targeting siRNA transfected HFFs at 8 hpi in (Fig 3C). This was further corroborated by a marked increase in expression of the IE ICP4 protein and in the E ICP8 protein levels at 24 hpi in HFFs transfected with TRIM22 siRNA and infected with HSV-1 7134 virus (Fig 3D, lane 4). We also observed an increase in TRIM22 protein levels in 7134 infection, consistent with TRIM22’s expression as an interferon-stimulated gene (Fig 3D, lane 3). Importantly, TRIM22 levels in 7134R infection were comparable to levels observed in mock infection, while levels of the known restriction factor IFI16, are reduced in 7134R infection (Fig 3E, lane 3, S4A–S4C Fig). This was consistent with the comparable TRIM22 protein levels observed in mock infection and in cells infected with HSV-1 *d*106 and HSV-1 KOS, both of which express the IE ICP0 protein (S4A Fig). These results indicated that the TRIM22-mediated inhibition of the HSV-1 ICP0-null virus is either at or prior to the level of IE gene transcription.

### Depletion of TRIM22 increases HSV-1 viral DNA replication and L gene expression

**Fig 4:**
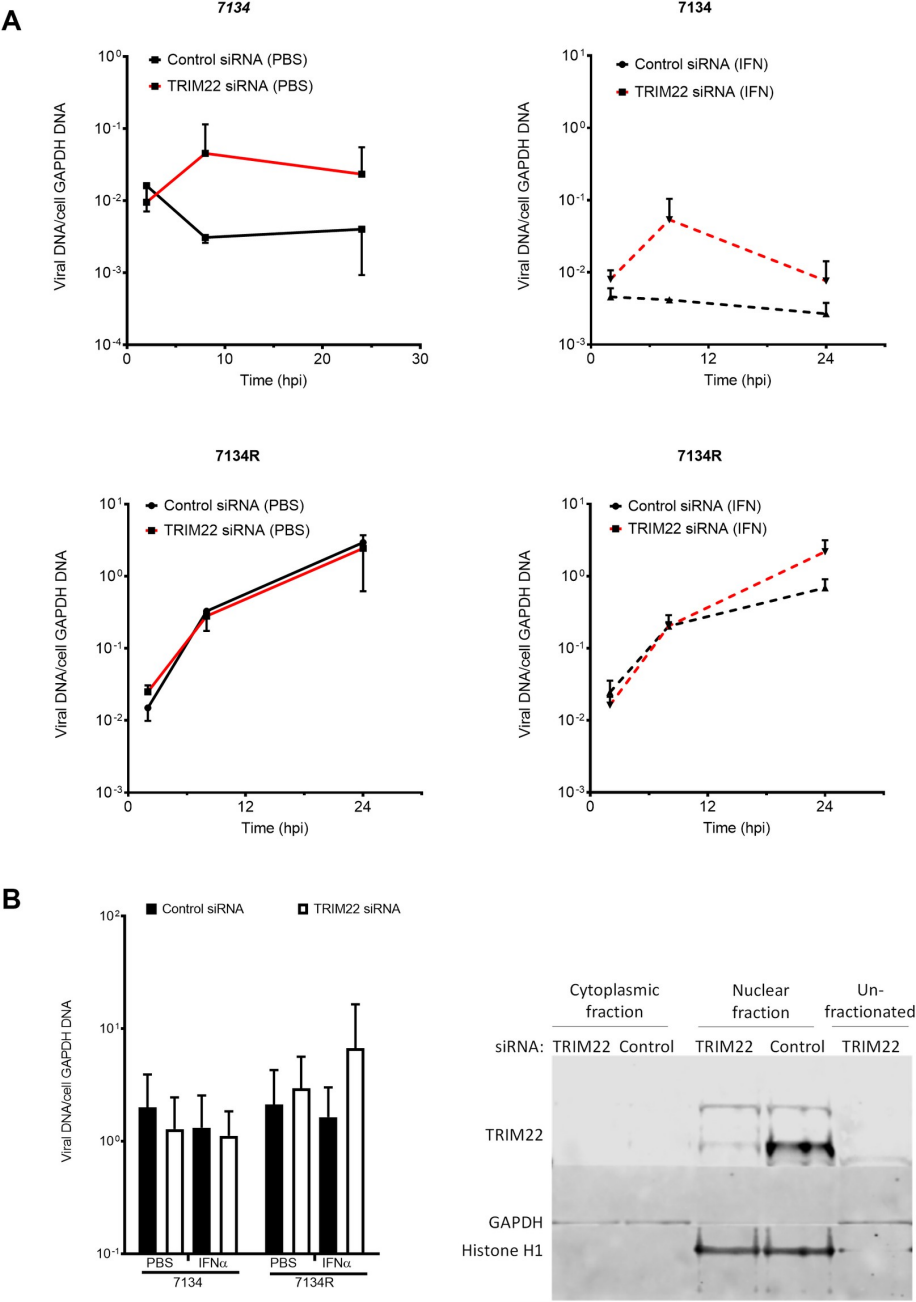
TRIM22 depletion increases viral DNA replication. HFFs transfected with siRNA pools specific for TRIM22 (red lines) or non-targeting siRNAs (black lines) and treated with PBS (solid lines) or hIFNα-2a (dashed lines) at 1000U/ml for 24 h were infected with HSV-1 ICP0-null (7134) (top panels) or a rescued virus (7134R) (bottom panels) at an MOI of 5. (A) Total cell-associated DNA was harvested at 2 hpi (n = 4), 8 hpi (n = 3), and 24 hpi (n = 2) and prepared for qPCR. Relative viral DNA levels were determined by normalizing *ICP8* vDNA levels to cellular *GAPDH* levels. (B) Left panel: DNA was extracted from the nuclear fractions of infected cells at 2 hpi and prepared for qPCR (n = 3). Right panel: The western blot shows the efficiency of fractionation qualitatively. Error bars represent standard errors of the means.

To determine whether the defect in IE and E gene expression was evident at the downstream stage of viral DNA (vDNA) synthesis, we measured vDNA levels in TRIM22-depleted and control-depleted HFFs. TRIM22 depletion increased vDNA synthesis of the 7134 virus in both PBS pre-treated and IFNα pre-treated HFFs, consistent with the increase in IE and E viral gene expression (Fig 4A). In addition, the TRIM22-mediated effect on vDNA synthesis was consistent with the increase in the expression levels of transcripts encoding components of the viral replication machinery: *U*_*L*_*5* (~3 fold), *U*_*L*_*8* (~4 fold), *U*_*L*_*9* (~4 fold), *U*_*L*_*30* (~3 fold), *U*_*L*_*42* (~6 fold) and *U*_*L*_*52* (~4 fold) (S5 Fig). Effects of TRIM22 on viral gene expression and vDNA replication were corroborated by late (L) viral gene expression as TRIM22 depletion increased *gC* transcript levels and protein levels relative to control-depleted HFFs (S6 Fig). The increase in viral IE gene expression was not due to altered nuclear entry of the virus, as TRIM22 depletion did not alter the number of 7134 virus genomes associated with the nuclear fractions at 2 hpi (Fig 4A, left panel). Western blots showed that cellular fractionation was effective, and TRIM22 was localized in the nucleus (Fig 4B, right panel). We also concluded that the TRIM22-mediated defect in HSV-1 replication was not entirely dependent on IFNα pre-treatment. Therefore, TRIM22-mediated defect in IE gene expression results in an inhibition of viral processes downstream of IE gene expression, including viral DNA synthesis, in a mechanism independent of nuclear entry.

### TRIM22 depletion reduces total histone occupancy and facultative heterochromatin on IE viral gene promoters

**Fig 5:**
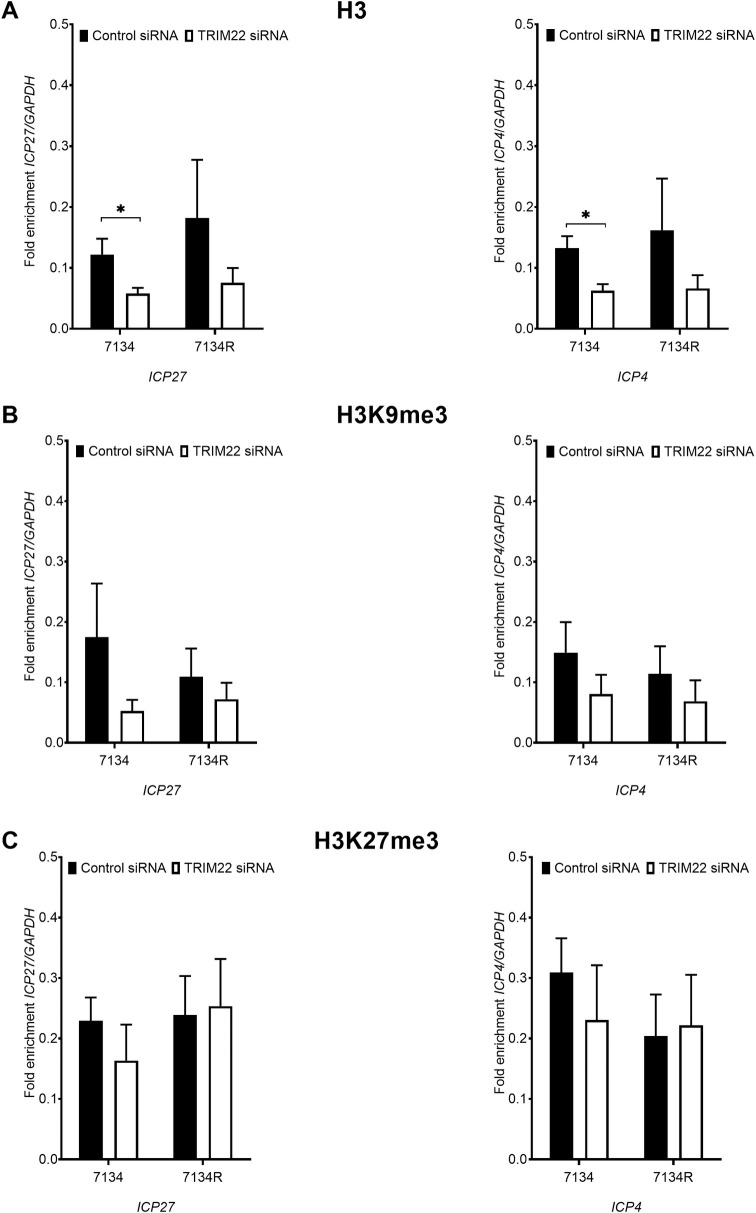
TRIM22 depletion reduces total histone H3 and facultative heterochromatin association with viral DNA. Control- or TRIM22-siRNA transfected HFFs were infected with HSV-1 ICP0-null (7134) or HSV-1 ICP0-rescued (7134R) viruses at an MOI of 5. ChIP was conducted on cell extracts prepared at 6 hpi with antibodies specific for histone H3 (n = 6) (A), the heterochromatin mark H3K9me3 (n = 3) (B), and the facultative heterochromatin mark H327me3 (n = 3) (C). Immunoprecipitated *ICP27* (left panels) and *ICP4* (right panels) promoter sequences were measured by qPCR and viral DNA sequences were normalized to immunoprecipitated *GAPDH* DNA. (*P<0.*05*, **P<0.*01* unpaired t-test).

Host cells silence foreign DNA upon nuclear entry by assembling heterochromatin onto free DNA [47–49]. In addition, this chromatin is modified by post-translational modifications such as H3K9me3 and H3K27me3, leading to compaction of chromatin to form heterochromatin, or by H3K9Ac and H3K4me3 post-translational modifications, leading to the more active euchromatin. To determine whether TRIM22 depletion affects viral chromatin, we conducted chromatin immunoprecipitation (ChIP) assays for total histone H3, the heterochromatin mark H3K9me3, the facultative heterochromatin mark H3K27me3, and the euchromatin marks H3K4me3 and H3K9Ac on the viral IE *ICP4* and *ICP27* gene promoters in TRIM22-depleted fibroblasts relative to control-depleted fibroblasts. TRIM22 depletion significantly reduced the total histone H3 immunoprecipitated on the viral *ICP27* and *ICP4* gene promoters in the 7134 virus infection (Fig 5A). In addition, TRIM22 depletion reduced the density of H3K9me3 and H3K27me3 immunoprecipitated on the *ICP27* and *ICP4* promoters in the 7134 virus infection, indicating that the viral DNA is not silenced to the same extent in TRIM22-depleted fibroblasts (Fig 5B and 5C). The reduced heterochromatin on viral DNA in TRIM22-depleted fibroblasts was consistent with the increase in viral IE gene expression observed in Fig 3. Although TRIM22 depletion did not significantly change the levels of euchromatin marks on *ICP27* and *ICP4* promoters, there was a trend towards increased density of H3K9Ac immunoprecipitated on the *ICP27* promoter in 7134 and 7134R virus infection under conditions of TRIM22 depletion (S7 Fig). Therefore, TRIM22 increases the loading of total histone H3 and heterochromatin marks on viral lytic gene promoters, consistent with TRIM22 promoting the epigenetic silencing of these genes.

### Functions of TRIM22 needed for restriction

The domains of TRIM22 have defined functions in the cell. The N-terminal RING domain has E3 ubiquitin ligase activity [50] and was reported to mediate anti-viral activity against hepatitis B virus (HBV) [51], encephalomyocarditis virus (ECMV) [35] and influenza A virus (IAV) [37]. The C-terminal B30.2/SPRY domain and the bipartite nuclear localization signal (NLS) are necessary for the nuclear localization of TRIM22 [44]. Therefore, we investigated whether these domains were necessary for TRIM22-mediated inhibition of HSV-1 by expression of various mutant forms of TRIM22.

**Fig 6:**
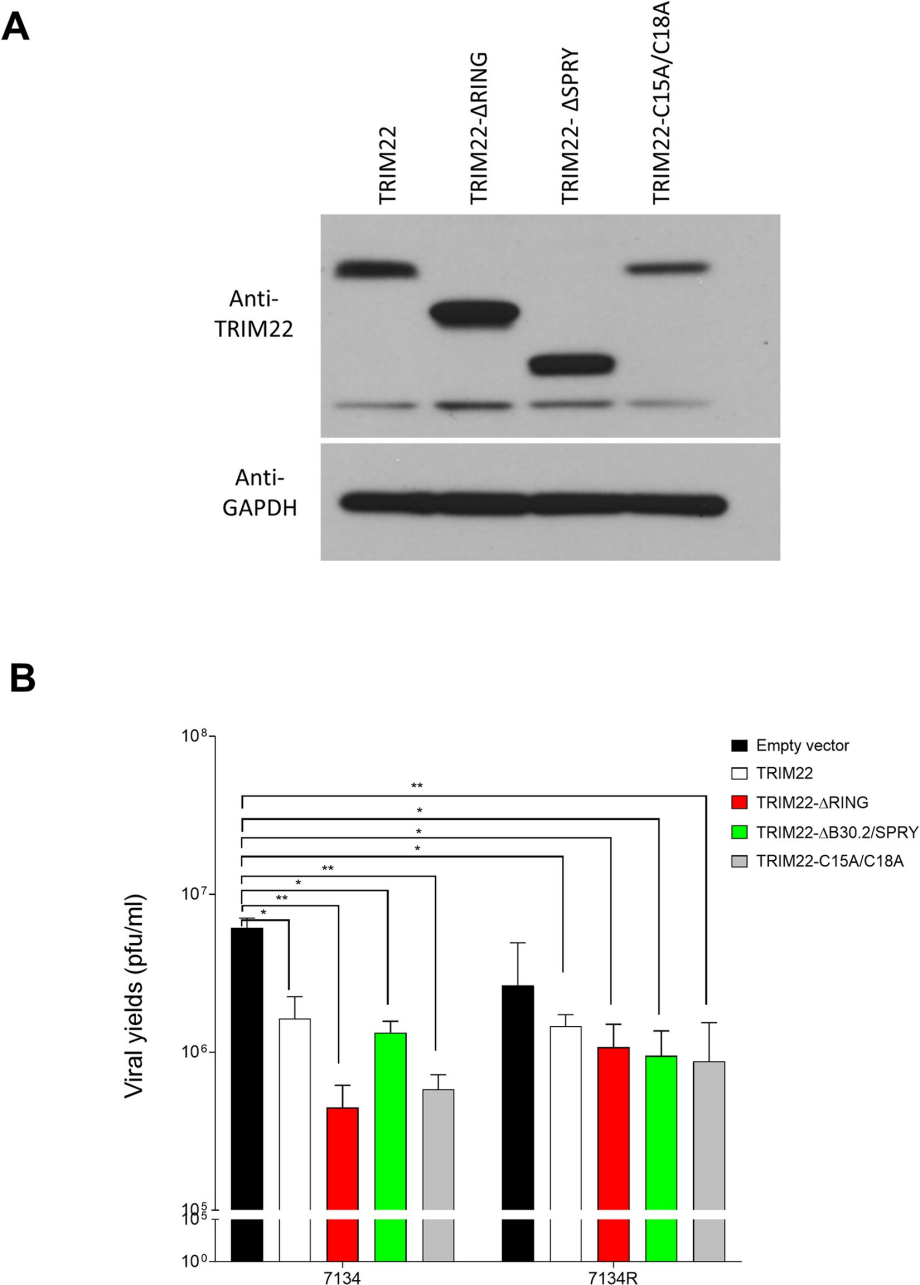
Role of TRIM22 domains in HSV-1 replication in stably-transfected cell lines. HeLa cells were transformed with an empty vector control (Empty vector) or vectors encoding full-length TRIM22 (TRIM22), the SPRY-domain deleted TRIM22 (TRIM22-ΔB30.2/SPRY), the RING-domain deleted TRIM22 (TRIM22-ΔRING), the E3 ubiquitin-ligase inactive form of TRIM22 (TRIM22-C15A/C18A). Cell lysates were analyzed with western blots probed for the TRIM22 and GAPDH as a loading control (A). These cells were infected at an MOI of 0.1 with 7134 or 7134R virus, and virus yields were measured at 48 hpi using plaque assays on U2OS cells (n = 3) (B). (*P<0*.05*, *P<0*.005**, Two-way ANOVA, multiple comparisons with Tukey’s corrections).

We generated stable HeLa cell lines by transformation with the following plasmids: empty vector or FLAG-tagged constructs encoding full-length TRIM22 (TRIM22), a SPRY-domain deleted TRIM22 (TRIM22-ΔB30.2/SPRY), a RING-domain deleted TRIM22 (TRIM22-ΔRING) and an E3 ubiquitin-ligase inactive form of TRIM22 (TRIM22-C15A/C18A) [44]. We were unable to generate stable cell lines with a TRIM22-ΔNLS mutant plasmid, apparently because it was toxic to cells. We observed that the expression levels of the TRIM22-ΔB30.2/SPRY and TRIM22-ΔRING constructs were higher than the protein levels of the full length TRIM22 construct, whereas the TRIM22-C15A/C18A construct was expressed at a lower level (Fig 6A). We then infected the stably transformed cells with the 7134 and 7134R viruses at an MOI of 0.1 and measured virus yields at 48 hpi. Cells stably expressing full-length TRIM22, TRIM22-ΔB30.2/SPRY, TRIM22-ΔRING and TRIM22-C15A/C18A all had reduced viral yields of the ICP0-null 7134 virus and the rescued 7134R virus, relative to cells stably transformed with the empty vector (Fig 6B). Therefore, multiple domains or an untested domain of the TRIM22 protein are capable of restriction of HSV-1 infection, and/or the mutant proteins showed a gain-of-function phenotype.

### TRIM22 may mediate part of the natural variation in susceptibility to HSV-1

**Fig 7:**
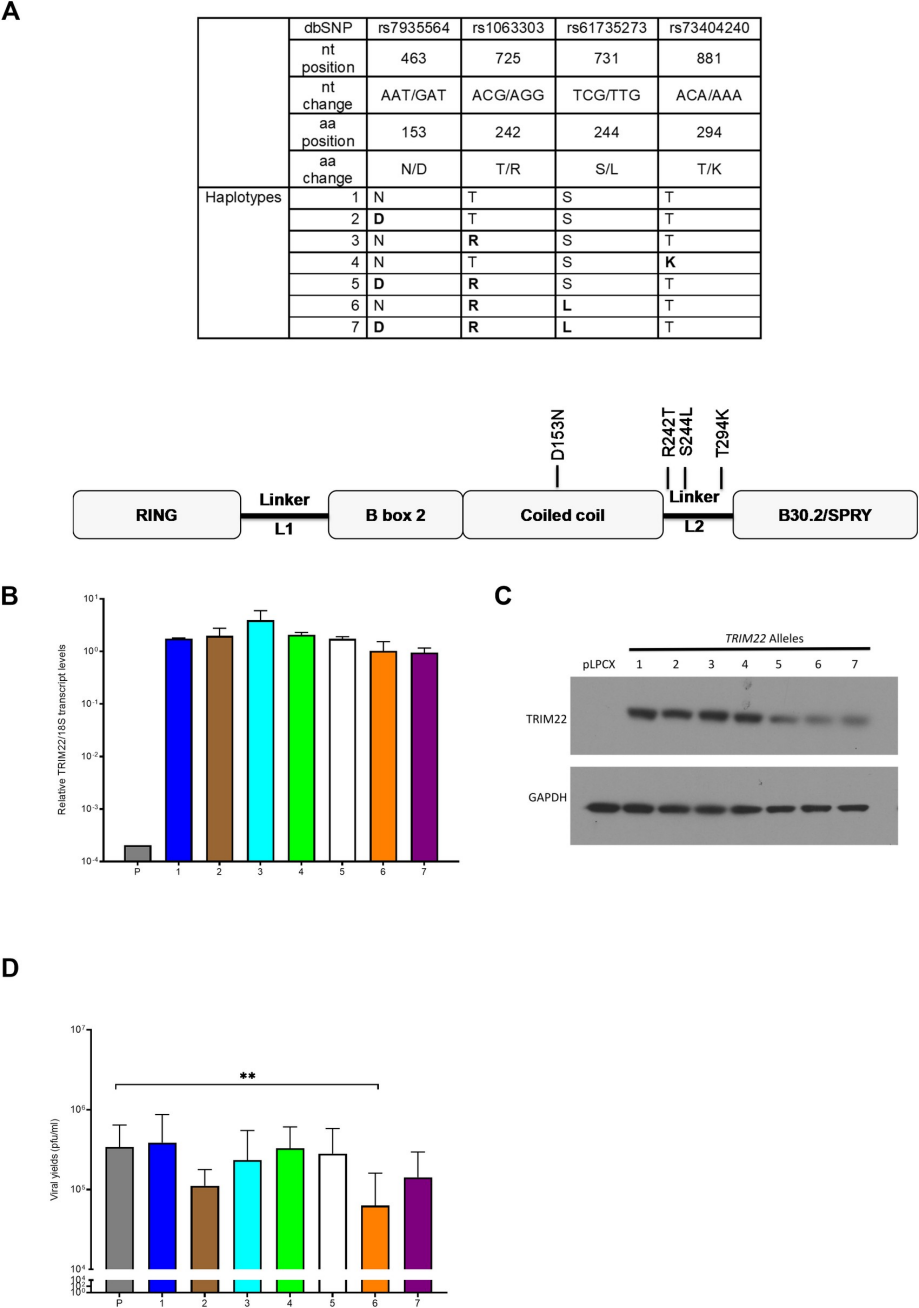
Human *TRIM22* alleles show variation in inhibition of HSV-1 replication. *TRIM22* cDNA sequencing was conducted on the human lymphoblastoid cell lines (LCLs) in the Caucasian and Yoruba cohorts generated in the HapMap project. A) Upper panel: The resulting combinations of SNPs in the *TRIM22* gene were labeled in the whole population. Lower panel: The schematic diagram shows the location of the amino acid substitutions when mapped to the predicted domain structure of the TRIM22 protein). HeLa cells transfected with an empty vector control (pLPCX) or the pLPCX vector with a TRIM22 haplotype insert (1, 2, 3, 4, 5, 6, and 7) and total cell-associated RNA was harvested at 8 hpi and prepared for qRT-PCR. (B) *TRIM22* transcripts were measured and normalized to 18S rRNA (n = 2). (C) Representative western blot showing total cell lysates probed for TRIM22 and GAPDH protein levels. (D) HeLa cells transfected with an empty vector control (pLPCX) or the pLPCX vector with a TRIM22 haplotype insert (1, 2, 3, 4, 5, 6, and 7) were infected with ICP0-null (7134) virus at an MOI of 0.1. Virus yields were determined 48 hpi with plaque assay on U2OS cells (n = 5). *P*<0.05*, *P<0*.005**, *P<0*.0002***,*P<0*.0001****) One-way ANOVA, multiple comparisons with Dunnett’s corrections).

The genetic variation in TRIM22-mediated inhibition of HSV-1 was defined by sequencing the *TRIM22* gene locus in B lymphoblastoid cell lines (B LCLs) generated from the Caucasian and Yoruba cohorts [43] in the HapMap project [52]. These studies identified the following *TRIM22* alleles in the populations studied encoding the following single nucleotide polymorphisms (SNPs) and the corresponding amino acid substitutions: rs7935564 A/G (N153D), rs1066303 G/C (R242T), rs61735273 C/T (S244L), rs73404240 C/A (T294K) [52] (Fig 7A, top panel). This allelic variation resulted in seven different *TRIM22* haplotypes (Fig 7A, top panel). These encoded amino acid substitutions are predominantly located in the linker L2 domain of the TRIM22 protein (Fig 7A, bottom panel). Previous studies had shown that polymorphisms in TRIM22 altered the protein’s ability to inhibit viruses [31–33,39]. Therefore, to define the effect of the allelic variation on HSV-1 replication, we transfected HeLa cells with the vectors encoding the different *TRIM22* haplotype forms [43] and infected the cells with HSV-1 7134 virus at a MOI of 0.1 pfu/cell to assess the role of the different *TRIM22* haplotypes in HSV-1 replication. Transfection of the different *TRIM22* haplotypes demonstrated similar levels of TRIM22 transcript and protein expression except for those of *TRIM22* haplotypes #5, #6 and #7, which showed reduced transcripts and protein levels (Fig 7, panels B-C).

We observed that transfection of all TRIM22 haplotypes except TRIM22 haplotype #1 showed reduced 7134 viral yields, but that the inhibition occurred to different extents (Fig 7D). Transfection of the *TRIM22* haplotype #5 did demonstrate lower levels of inhibition than those observed in the over-expression studies in Fig 2B, likely due to the lower levels of TRIM22 expression relative to GAPDH in this set of experiments (Figs 7C and 2C). We observed that transfection with TRIM22 haplotypes #2 and #6 had the greatest effect on 7134 viral yield inhibition relative to empty vector transfection (Fig 7D). The absence of multiple single nucleotide polymorphisms common to both *TRIM22* haplotypes #2 and #6 suggested there are several factors including TRIM22’s protein structure and interacting partners that help define the activity of the different *TRIM22* alleles in HSV-1 infection. Most importantly, these results raised the idea that variation in the *TRIM22* gene locus could play a role in varying levels of individual susceptibility to HSV-1 infection in humans.

### TRIM22-mediated inhibition extends to other herpesviruses

**Fig 8:**
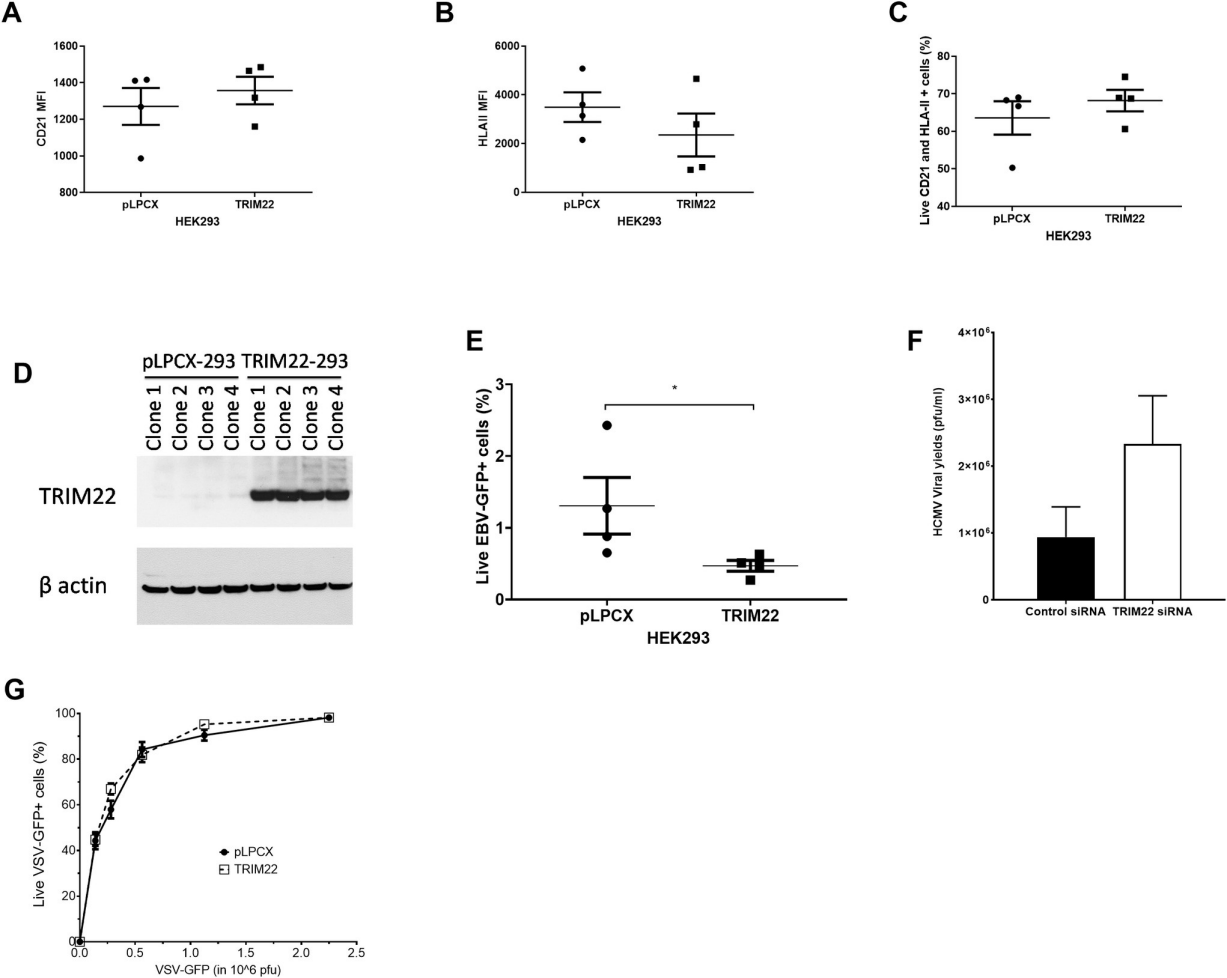
TRIM22 reduces replication of a β- and a γ-herpesvirus. (A-E) EBV. HEK293 cells stably transfected with CD21 and HLA-II, and either the empty vector (pLPCX) or the full-length TRIM22 vector (TRIM22) were infected with EBV-GFP at 17GRU. CD21 MFI (A) and HLA-II MFI (B) and percentage of live cells as determined by counter-staining with aqua-amine that are CD21 and HLA-II double-positive (C) were quantified by flow cytometry. Whole cell lysates from stably transfected empty vector (pLPCX) or TRIM22 transfected (TRIM22) single-cell clones were probed for TRIM22 and GAPDH by immunoblotting (D). Percentage of live EBV-GFP cells in pLPCX and TRIM22 cells were quantified at 5 dpi (n = 4) (E). (F) HCMV. HFFs were transfected with siRNA pools specific for TRIM22 or non-targeting siRNAs. Control- or TRIM22- siRNA transfected HFFs were infected with HCMV Ad 169 at an MOI of 1 and virus yields were measured by plaque assays on HFFs at 72 hpi (n = 3). (G) VSV. HEK293 cells stably transduced with either the empty vector (pLPCX) or the full-length TRIM22 vector (TRIM22) were infected with VSV-GFP at a range of dilutions from 0 to 2.5x10^6^ pfu/ml, and assessed by flow cytometry for live, infected cells at 16 hpi (n = 3). (*P*<0.05*, Mann-Whitney t-test).

Previous reports demonstrated that TRIM22 inhibits a diverse range of viruses, including RNA viruses such as HIV-1, EMCV, IAV, and a DNA virus, HBV [34–37]. To test whether TRIM22 was capable of inhibiting other herpesviruses besides the α-herpesvirus, HSV-1, we investigated whether TRIM22 could inhibit a γ-herpesvirus, Epstein-Barr virus (EBV). In fact, there is evidence that the latent membrane protein 1 (LMP1) of EBV promotes an anti-viral cellular state by upregulating ISGs such as TRIM22 to prevent super-infection [41]. Therefore, we measured the efficiency of EBV-GFP infection in HEK293 cells expressing the EBV receptors, CD21 and HLA-II, after stable transfection with the empty vector (pLPCX) or full-length TRIM22 (TRIM22). Transformation of cells with *TRIM22* did not significantly alter the expression of CD21 or HLA-II or the percentage of CD21^+^ HLA-II^+^ transduced HEK293 cells (Fig 8A, 8B and 8C). TRIM22 expression was confirmed by immunoblotting in multiple single-cell clones and compared to endogenous levels in cells expressing the empty control pLPCX vector (Fig 8D). We observed significantly fewer EBV-GFP-positive cells in TRIM22-transfected cells relative to pLPCX-transfected cells (Fig 8E). These results implied that TRIM22 reduces the efficiency of EBV-GFP infection. We also measured HCMV viral yields at 72 hpi in control-depleted versus TRIM22-depleted HFFs. TRIM22 depletion significantly increased HCMV viral yields by approximately 4-fold relative to the control depleted cells (Fig 8F). Therefore, TRIM22 also inhibited HCMV replication, although to a lesser extent than observed for HSV-1 using the same primary human fibroblasts and siRNAs. Interestingly, TRIM22 transfection did not inhibit infection of an RNA virus that replicates in the cytoplasm, VSV-GFP (Fig 8G). In conclusion, the TRIM22-mediated viral inhibition was not limited to the α-herpesviruses, and TRIM22 inhibited other DNA viruses that replicate in the nucleus, including the β- and γ- herpesviruses.

## Discussion

### TRIM22 acts as a restriction factor of HSV-1 by epigenetic regulation of viral genes

HSV-1 restriction in the nucleus has been ascribed previously to components of ND10 bodies in the nucleus consisting of TRIM19/PML, hDaxx, Sp100, ATRX; E3 SUMO ligases such as PIAS 1 and PIAS 4; DNA repair proteins such as RNF8 and RNF168 [19,53,54], reviewed in [55], and to nuclear IFI16 [56,57]. We add TRIM22 to this list by demonstrating that TRIM22 is necessary and sufficient for part of the restriction of HSV-1 inhibition. TRIM22 depletion in fibroblasts rescued the replication of an ICP0^-^ virus and to a smaller extent, an ICP0^+^ virus. The inhibition of both the ICP0^-^ and ICP0^+^ viruses by TRIM22 over-expression studies in HeLa cells further corroborated this finding. TRIM22-mediated inhibition of HSV-1 in HeLa cells was not observed at high MOIs, reflecting an MOI-dependent effect where the virus is capable of overcoming the intrinsic restriction at high MOIs in HeLa cells. This MOI-dependent effect on viral restriction is a common feature of intrinsic immunity and was previously reported in the rhesus macaque TRIM5α-mediated restriction of HIV-1 [58,59].

TRIM22 was shown previously to inhibit viruses by various mechanisms, including degradation of viral proteins as seen in EMCV [35] and IAV [37], transcriptional repression [36,60] and inhibition of viral assembly [34] as seen in HIV-1, and prevention of viral core promoter activity as seen in HBV [38]. We discovered that the TRIM22-mediated inhibition of HSV-1 replication was due to a defect in viral IE gene expression after nuclear entry of viral genomes, with downstream effects on E gene expression, viral DNA synthesis and L gene expression. Though the initial reduction in IE gene expression is limited, this effect is likely amplified downstream in E and L gene expression resulting in the eventual changes in virus yield. There may be additional TRIM22-mediated effects on the virus downstream of early gene expression, which may contribute to the eventual loss of progeny production. A possible mechanism of inhibition explored here is that TRIM22 increases histone occupancy, and, in parallel, heterochromatin marks on viral DNA. This is consistent with previously findings that total H3 density trends with heterochromatin marks [61]. Chromatin modification as a means of viral inhibition has been directly attributed to IFI16 [57], and ATRX in concert with hDaxx to a smaller extent, as a chromatin remodeling complex [14].

### TRIM22 restricts viruses that replicate in the nucleus

The importance of nuclear-specific inhibition of HSV-1 was evident from the ability of PML, hDaxx, Sp100, and ATRX in ND10 bodies [53,54], and IFI16 [57], to inhibit steps of HSV-1 replication in the infected cell nucleus. PML has also been demonstrated to colocalize with endogenous TRIM22 in Hep-2 cells treated with IFNγ [62]. In addition, the endogenous nuclear localization of TRIM22 in primary fibroblasts, where the inhibitory effect of TRIM22 was observed, lends further evidence to the case for recognition and restriction of foreign DNA in the nucleus. This report expands the antiviral specificity of TRIM22 to include the herpesviruses.

The TRIM22-mediated antiviral mechanisms of inhibition of other viruses can be attributed predominantly to its E3 ubiquitin ligase activity located in the N-terminal RING domain of the protein [35,37,38]. In the case of HSV-1, the localization of TRIM22 to nuclear punctate structures [44] and the E3 ubiquitin ligase activity needs to be further explored as the absence of the RING domain did not affect TRIM22-mediated restriction of HSV-1. TRIM22 was also reported to co-localize to TRIM19/PML bodies endogenously in a RING-domain independent manner [62], suggesting that the nuclear localization of TRIM22, rather than the RING domain mediated E3 ubiquitin ligase activity, is more crucial to the restriction activity of TRIM22.

The SPRY domain of TRIM22 has been implicated in the protein’s subcellular localization as others have observed: (i) the absence of B30.2/SPRY domains in a transfection system prevents the formation of nuclear punctate structures [44]; and (ii) the different subcellular localization of human and rhesus TRIM22 proteins has been attributed to the positively-selected SPRY domain [45]. In addition, the absence of the SPRY domain was shown to abrogate the TRIM22-mediated inhibition of HBV; this was primarily due to the cytoplasmic localization of this particular construct [38]. We observed that mutant forms of TRIM22 defective for each of the known domains can still inhibit HSV-1 ICP0- virus replication, so multiple mechanisms may exist or some of the mutant forms may have a gain-of-function phenotype. Further finer mutational analysis is needed to define the domains of TRIM22 needed for inhibition of HSV-1.

Therefore, TRIM22 can inhibit viruses that replicate in the nucleus: e.g., IAV [37], HBV [38], and in this study herpesviruses including members of the α-, β- and γ-herpesviruses, while it is unable to restrict certain viruses that replicate in the cytoplasm such as the RNA virus, VSV. On the other hand, TRIM22 also inhibits EMCV, a positive strand RNA virus that replicates in the cytoplasm [35]. This is consistent with the activity of other restriction factors that localize to and inhibit pathogens in specific cellular compartments.

### Co-evolution of the *TRIM5* and *TRIM22* gene loci with herpesviruses

The *TRIM5* and *TRIM22* gene neighbors evolved under positive selection in hominoids and Old World monkey species, such that they were positively selected in different species [28]. In fact, the rhTRIM5α inhibits HIV-1 greatly [58], whereas TRIM22 in humans shows a greater degree of HIV-1 inhibition [34] than human TRIM5 [28].This is a similar pattern to what is observed in the herpesviruses. We previously found that the rhTRIM5α protein was capable of cross-species restriction of human HSV-1 [27], and we report here that the human TRIM22 protein also has an ability to restrict human HSV-1. In contrast, the herpesviral restriction by human TRIM5 is to a much smaller extent [27].

Furthermore, Sawyer et al. [28] have demonstrated that the *TRIM22* gene locus in humans has a high ratio of non-synonymous to synonymous nucleotide substitutions (d_N_/d_S_), reflecting a signature of positive genetic selection. This evolutionary pressure potentially resulted in allelic diversification, and we also demonstrate that the seven different haplotypes of TRIM22 show varying degrees of HSV-1 inhibition. Furthermore, some of these polymorphisms in TRIM22 have been shown to vary in the degree of viral inhibition [31,32,63]. The nucleotide variation in *TRIM22* is reported as the following SNPs in the NCBI database: rs10838543 T/C, rs7935564 A/G and rs1063303 C/G [39]. In the context of HBV infection, Han-Chinese individuals homozygous for *TRIM22*-364 C/C genotype were associated with chronic HBV infection [38]. In the context of HIV-1 inhibition, transfection of *TRIM22* alleles homozygous for G at the SNPs rs7935564 and rs1063303 in 293T cells show a decreased capacity to inhibit HIV-1 transcription [31]. Consistent with this, HeLa cells transfected with the TRIM22 variant homozygous for G at rs1063303 did not demonstrate an inhibition in HIV-1 Gag production [34]. In contrast, we observed that HeLa cells transfected with the TRIM22 variants homozygous for G at the SNPs rs7935564 and rs1063303 had reduced 7134 virus yields, consistent with the capability of these variants for inhibition of ICP0-null virus replication. Consequently, we speculate that the dichotomy of the *TRIM5* and *TRIM22* gene loci is pathogen-driven and due to selection pressures from herpesviruses, in addition to the previously known lentiviruses.

### HSV-1 mediated escape of TRIM22-mediated inhibition

We attribute the ability of the wild-type virus to overcome TRIM22-mediated inhibition, at least in part, to the viral IE protein, ICP0. This protein is crucial to alleviating a majority of the intrinsic- and the Type I IFN mediated- immune responses: i.e., (i) it disrupts PML bodies at sites of viral replication [64], preventing their intrinsic inhibition; (ii) it sequesters phosphorylated IRF3 from the nucleus [20,65], thus preventing its activity as a transcription factor; and (iii) it degrades the nuclear viral DNA sensor and intrinsic resistance factor, IFI16, thus preventing detection and silencing of viral DNA [16]. Therefore, the finding that ICP0 rescued most of the intrinsic block by TRIM22 is consistent with previous studies. However, viruses with ICP0 such as the 7134R, HSV-1 KOS and HSV-1 *d*106 viruses did not significantly reduce TRIM22 protein levels. Surprisingly, ICP0 does not seem to promote the degradation of TRIM22, and it remains to be determined how the virus evades the intrinsic restriction activity of TRIM22 and whether there are other herpesviral factors that antagonize TRIM22.

In conclusion, the key features of restriction factors are that they: (i) are dominantly acting and show cell-autonomous mechanisms of anti-viral activity from a genetic standpoint; (ii) are germline-encoded, expressed constitutively, and often interferon (IFN)-inducible; (iii) demonstrate atypical mechanisms of viral inhibition; and (iv) show hallmarks of positive genetic selection (high d_N_/d_S_ ratios) reflecting host-pathogen co-evolution [1]. Consistent with previous studies, we show that TRIM22 encompasses these features, and, in this study, we report that TRIM22 is a novel restriction factor of HSV-1. In addition, TRIM22 is elevated in epithelial cells surrounding a herpes genital lesion, showing that it may function as an ISG to protect those cells *in vivo* [66]. Overall, the properties of TRIM22 are those of a restriction factor, and although chromatin modification is one mechanism of action, the detailed mechanisms and the intracellular location of TRIM22 mediated repression of viral DNA remain to be further explored.

## Materials and methods

### Cell culture and viruses

HFF (Catalog number: CRL-1634), U2OS, HeLa and HEK293 cells were obtained from the American Type Culture Collection. HFFs were cultured as described previously [16]. U2OS and HeLa cells were grown in DMEM supplemented with 5% (vol/vol) heat-inactivated fetal bovine serum (FBS) and 5% (vol/vol) heat-inactivated bovine calf serum (BCS).

HEK293 cells stably transduced with CD21 and HLA-II were provided by Joyce Fingeroth (University of Massachusetts Medical School) and cultured in selection media as described previously [67]. The ICP0-null (7134) and rescued 7134R viruses [68] were grown and titrated on U2OS cells in parallel [69]. The HSV-1 *d*109 and *d*106 viruses were propagated and titrated on FO6 cells and E11 cells, respectively [70]. HCMV AD169 was a kind gift from the Coen laboratory, and it was propagated as described previously [71].

### Virus infections

HSV-1 infections were conducted as described previously [57]. HSV-1 was diluted in PBS solution containing 0.1% glucose and 1% heat-inactivated BCS. Cells were infected at the stated MOI of either 0.1 or 5 for 1 h at 37°C, washed twice with PBS solution, and overlaid with DMEM containing 1% heat-inactivated BCS. Infected cells were incubated at 37°C for the indicated length of time of 48 h at the low MOI of 0.1 or 24 h at the high MOI of 5. For ChIP experiments testing the effects of inhibiting viral DNA synthesis, sodium phosphonoacetate (PAA) was added to the medium at 200 μg/ml along with 10 mM HEPES at the time of infection and maintained in the medium after infection until the cells were harvested at the indicated time points as described previously [72].

HCMV infections were conducted as described previously [73]. Briefly, HCMV AD169 stocks were diluted in DMEM containing 10% FBS and cells were infected at an MOI of 1 for 1 h at 37°C and washed twice with PBS solution. After incubation for 1 h at 37°C, the inoculum was removed and replaced with 1 mL of complete DMEM containing 10% fetal bovine serum (FBS). Infected cells were incubated at 37°C for 72 h. The medium from each well (virus supernatant) was taken from the cells and stored at −80°C until required. Dilutions of the virus supernatant were titrated simultaneously onto fresh monolayers of HFF cells to determine virus titers.

EBV-GFP stocks were prepared from a cell line [74] kindly provided by Wolfgang Hammerschmidt, the virus was diluted in DMEM containing 10% FBS, and cells were infected for 5 days at 37°C as described previously [67]. Briefly, cells were seeded at a density of 1 x 10^6^ in triplicate and washed in PBS. Washed cells were incubated with EBV-GFP virus for 1 h at 37°C. Cells were then washed three times in PBS and infection was allowed to proceed at 37°C in a six well plate.

### siRNA transfections

Double-stranded *TRIM22-*specific, and non-target control siRNAs were purchased from Dharmacon. The pooled siRNAs were transfected into HFFs using the DharmaFECT 2 transfection reagent (Dharmacon) at a final concentration of 50nM according to manufacturer’s instructions. Cells were split into two separate wells at 72 h post transfection (hpt) and transfected again as described earlier [57]. Cells were infected after another 72 hpt. The siRNA was replaced 24 hpt with incubation media in both rounds of transfection, and cells were assayed for TRIM22 or 18S levels by qRT-PCR and immunoblot at 72 hpt after the second round of transfection.

### Plasmids and DNA transfection

The TRIM22 cDNAs were prepared from RNA extracted from B lymphoblastoid cell lines from the HapMap project and cloned into the pLPCX vector backbone (Clontech) [43]. Sequences of the genes are available in GenBank (NM_006074). The plasmids encoding the TRIM22 constructs with mutationally altered domains were a kind gift from Dr. Valerie Lin [44]. HeLa cells were plated and transfected with the Effectene reagent according to the manufacturer’s instructions. Transfected cells were either treated with PBS or hIFNα-2a at 24 hpt and infected 24 h post treatment as described earlier in the Materials and Methods.

### Cellular RNA analysis by qPCR

Cellular RNA levels were measured as described previously [57]. Briefly, total RNA was extracted using the Qiagen RNeasy Kit and DNase treated using the DNA-free kit (Ambion). Equal amounts of RNAs were reverse-transcribed and quantified by real-time qPCR by using the Fast Power SYBR Green PCR master mix and Step One PCR sequence detection system (Applied Biosystems). qPCR reactions were carried out in duplicate, and relative copy numbers were determined by comparison with standard curves. Mock reverse-transcribed samples were included as negative controls. Transcript levels were normalized to 18S rRNA levels and made relative to mock-infected samples. Experiments were conducted three times, and the values were averaged. A list of primer sequences used is provided in S1 Table.

### Nuclear DNA analysis

Nuclei from 7134- or 7134R-infected cells were isolated using the NE-PER Nuclear and Cytoplasmic Extraction Kit (Thermo-Scientific), and DNA was harvested from these nuclei by using a Qiagen DNeasy blood and tissue column kit. Viral DNA levels were determined by qRT-PCR using the Power SYBR Green PCR master mix and a Prism 7300 sequence detection system (Applied Biosystems) as described previously [57]. PCR reactions were carried out in triplicate, and relative copy numbers were determined by comparison with standard curves. Viral DNA was normalized to cellular *18S* levels. A list of primer sequences used is provided in S1 Table.

### Western blots

Cells were lysed in NuPAGE LDS Sample Buffer, and proteins were resolved on NuPAGE 4% to 12% Bis-Tris gels (Invitrogen) as described previously [57]. Proteins were transferred overnight to nitrocellulose membranes and blocked with 5% milk in PBS solution containing 0.1% Tween-20 (PBS-T). Membranes were probed with primary antibody at 4°C overnight, washed with PBS-T and incubated in secondary antibody for 1 h at room temperature. Western blots were developed using the Super Signal Pico Chemiluminescence substrate. A list of antibodies and their dilutions is provided in S2 Table.

### Flow cytometry

Infected cells were trypsinized, collected by centrifugation and resuspended in 1 x phosphate-buffered saline (PBS) solution containing LIVE/DEAD Fixable Aqua Dead Cell Stain (Life Technologies) for 20 min at room temperature in the dark. Cells were washed twice in PBS and resuspended in 4% formaldehyde for 15 min at 4°C [67]. Cells were read using a LSR II and live cells were gated for using an Aqua-Amine negative gate after exclusion of doublets on forward and side scatter axes. GFP expressing cells were imaged using filters set for FITC with an excitation wavelength of 488 nm resulting in emission at 507 nm as described [75]. The GFP+ gate was defined on mock-infected cells. Data analysis was performed using FlowJo (version 8) software and graphs were constructed by using Graph Pad Prism software [57].

### Chromatin immunoprecipitation

HFFs were transfected with siRNAs as described earlier and after the first round of transfection, 5.5x10^5^ cells were plated in 60mm dishes and transfected for the second round of transfection. Cells were infected at 72 hpt, and the ChIP protocol was conducted as described previously [57]. Prior to immunoprecipitation, a 10μl aliquot of the sample was reverse cross-linked as described previously [57], and DNA was extracted and analyzed by gel electrophoresis in a 1% gel to ensure DNA fragments are ~500bp in length. Immunoprecipitation (IP) reactions were conducted as described previously [72]. Briefly, IP reactions contained 50 μg of chromatin diluted 10-fold in ChIP dilution buffer (150 mM NaCl, 10 mM Na2HPO4, 2 mM EDTA, 1.1% Triton, 0.1% SDS). From each immunoprecipitation reaction, 1% of the chromatin was removed and reserved for input measurements. Immunocomplexes were formed by overnight incubation at 4°C with antibody as follows: with 2.5μg of anti-histone H3 IgG (Abcam) and anti-histone H3K9me3 (Active Motif), or normal rabbit IgG (Millipore, 12–370) as a negative control. Antibody complexes were captured with 20 μl Magna ChIP protein A magnetic beads (Millipore) by incubation with IP samples at 4°C for 2 h. Antibody complexes were washed 3x with ChIP dilution buffer containing 0.1% SDS and 1 mM PMSF, 3x with lithium chloride wash buffer (50 mM HEPES, pH 7.5, 500 mM LiCl, 1 mM EDTA, 1% NP-40, 0.7% sodium deoxycholate, 1 mM PMSF), and once with Tris-EDTA pH 8.0 buffer. Complexes were eluted from beads twice with the addition of 90 μl of elution buffer (1% SDS, 0.1 M NaHCO3) for 10 min at 65°C. Formaldehyde cross-linking was reversed by addition of NaCl to a concentration of 200 mM and incubation for 30 min at 95°C, and the DNA was purified and isolated by treatment with 1 μg of RNase A (Ambion) at 37°C for 1 h, proteinase K at 45°C for 2 h, and use of the QIAquick PCR Purification kit (Qiagen) according to the manufacturer’s instructions.

## Supporting information

S1 Fig TRIM22 expression in different cell types. RNA was prepared for qRT-PCR from transformed cells (293, 293T, HeLa, U2OS) or primary cells (HFFs, normal oral keratinocytes (NOKs)) or HeLa cells transfected with the empty vector, pLPCX (P) or the vector encoding full-length TRIM22 (T). The levels of *TRIM22* transcripts were measured and normalized to *18S* rRNA (n = 3). (*P<0*.0001**** One-way ANOVA, multiple comparisons with Tukey’s corrections)
(TIF)

S2 Fig Overexpression of TRIM22 does not reduce HSV-1 replication at a high MOI. HeLa cells transfected with an empty vector control (pLPCX) or a vector with a TRIM22 insert (TRIM22) were treated with PBS or hIFNα-2a at 1000U/ml for 24 h. Transfected cells were infected with ICP0-null (7134) or rescued virus (7134R) at an MOI of 5. (A) Total cell-associated RNA was harvested at 3 hpi and prepared for qRT-PCR. *TRIM22* transcripts were normalized to 18S rRNA. (B) Virus yields at 24 hpi were determined with plaque assays on U2OS cells (n = 1).
(TIF)

S3 Fig Overexpression of TRIM22 inhibits HSV-1 replication in the first round of infection. HeLa cells transfected with an empty vector control (pLPCX) or a vector with a TRIM22 insert (TRIM22) were treated with PBS or hIFNα-2a at 1000U/ml for 24 h. Transfected cells were infected with ICP0-null (7134) or rescued virus (7134R) at an MOI of 0.1. (A) Total cell-associated RNA was harvested at 4 hpi and 8hpi and prepared for qRT-PCR. *ICP27* transcripts were normalized to 18S at 4 hpi (A) and at 8 hpi (B). *TRIM22* transcripts were normalized to 18S rRNA at 4 hpi (C) and at 8 hpi (D) (n = 1).
(TIF)

S4 Fig ICP0 does not promote the degradation of TRIM22. (A) Whole cell lysates from mock-infected or infected with HSV-1 *d*106, *d*109, or wildtype KOS viruses (MOI = 5) were probed for ICP0 and TRIM22 at 2 hpi, 6 hpi, and 12 hpi. (B) Whole cell lysates from control-depleted or TRIM22-depleted HFFs that were either mock-infected or infected with 7134 or 7134R viruses (MOI = 5) were probed for TRIM22, GAPDH and the viral proteins ICP27, ICP8, gC, and TRIM22 at 4 hpi and 8 hpi. (C) Whole cell lysates from control-depleted or TRIM22-depleted HFFs that were pre-treated with either PBS or hIFNα (1000U/mL for 24 h) were either mock-infected or infected with 7134 or 7134R viruses (MOI = 5) were probed for TRIM22, GAPDH, and the viral proteins ICP27, ICP8, and gC at 4 hpi and 8 hpi.
(TIF)

S5 Fig TRIM22 depletion increases expression of components of viral replication machinery. TRIM22-depleted or control-depleted HFFs were treated with PBS or hIFNα-2a at 1000U/ml for 24 h and infected with HSV-1 ICP0-null (7134) or a rescued virus (7134R) at an MOI of 5. Total cell-associated RNA was harvested 8 hpi and transcript levels of *U*_*L*_*5* (A), *U*_*L*_*8* (B), *U*_*L*_*9* (C), *U*_*L*_*30* (D), *U*_*L*_*42* (E), and *U*_*L*_*52* (F) were measured by qRT-PCR. The transcript levels were normalized to *18S* rRNA (n = 3). Fold differences due to TRIM22 depletion are shown above corresponding bars.
(TIF)

S6 Fig TRIM22 depletion increases L gene expression. HFFs were transfected with siRNA pools specific for TRIM22 or non-targeting siRNAs and were treated with PBS or hIFNα-2a at 1000U/ml for 24 h and infected with HSV-1 ICP0-null (7134) or a rescued virus (7134R) at an MOI of 5. (A) Total cell-associated RNA was harvested at 4 hpi (left panel) and 8 hpi (right panel) and prepared for qRT-PCR. *gC* transcripts were measured and normalized to 18S rRNA levels (n = 2) (A). Fold differences due to TRIM22 depletion are shown above corresponding bars. (B) Whole cell lysates were collected at 4 hpi and 8 hpi and the representative western blot shows gC, TRIM22 and GAPDH protein levels.
(TIF)

S7 Fig TRIM22 depletion increases euchromatin association with viral DNA. Control- or TRIM22-siRNA transfected HFFs were infected with HSV-1 ICP0-null (7134) or HSV-1 ICP0-rescued (7134R) viruses at an MOI of 5. ChIP was conducted on cell extracts prepared at 6 hpi with antibodies specific for the euchromatin mark H3K9Ac (n = 3) (A). Immunoprecipitated ICP27 (left panel) and ICP4 (right panel) promoter sequences were measured by qPCR and viral DNA sequences were normalized to immunoprecipitated GAPDH DNA.
(TIF)

S1 Table List of primer sequences used in the study. (PDF)

S2 Table List of antibodies and their dilutions used in the study. (DOCX)
