# Supplementary figures and images for: Tripartite Motif 22 (TRIM22) protein restricts herpes simplex virus 1 by epigenetic silencing of viral immediate-early genes
Source: PLoS Pathog. 2021 Feb 1;17(2):e1009281. doi: 10.1371/journal.ppat.1009281 (PMC7877759; doi:10.1371/journal.ppat.1009281)

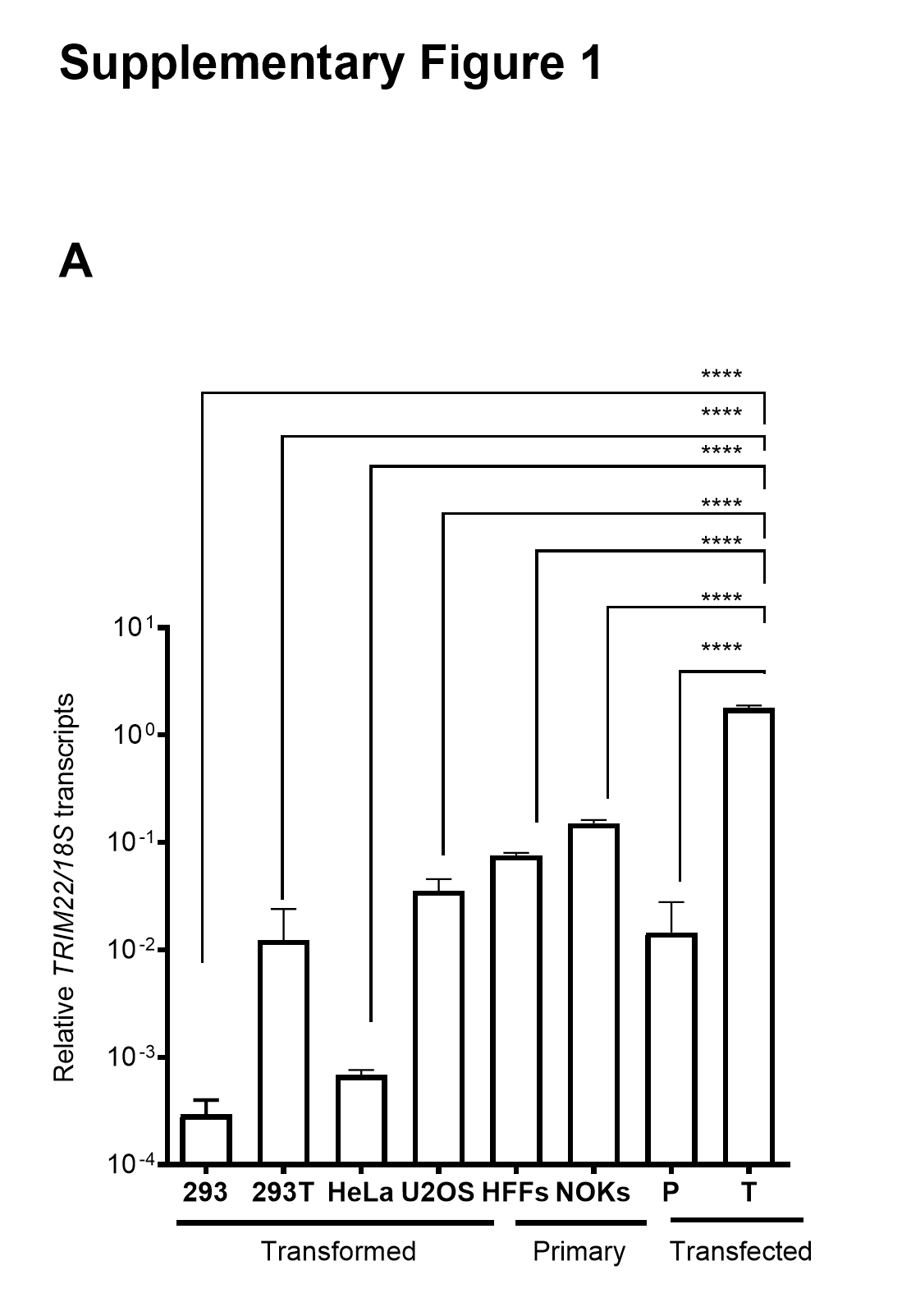

Supplement: S1 Fig — RNA was prepared for qRT-PCR from transformed cells (293, 293T, HeLa, U2OS) or primary cells (HFFs, normal oral keratinocytes (NOKs)) or HeLa cells transfected with the empty vector, pLPCX (P) or the vector encoding full-length TRIM22 (T). The levels of TRIM22 transcripts were measured and normalized to 18S rRNA (n = 3). (P<0.0001**** One-way ANOVA, multiple comparisons with Tukey’s corrections) (TIF) [file ppat.1009281.s001.tif]

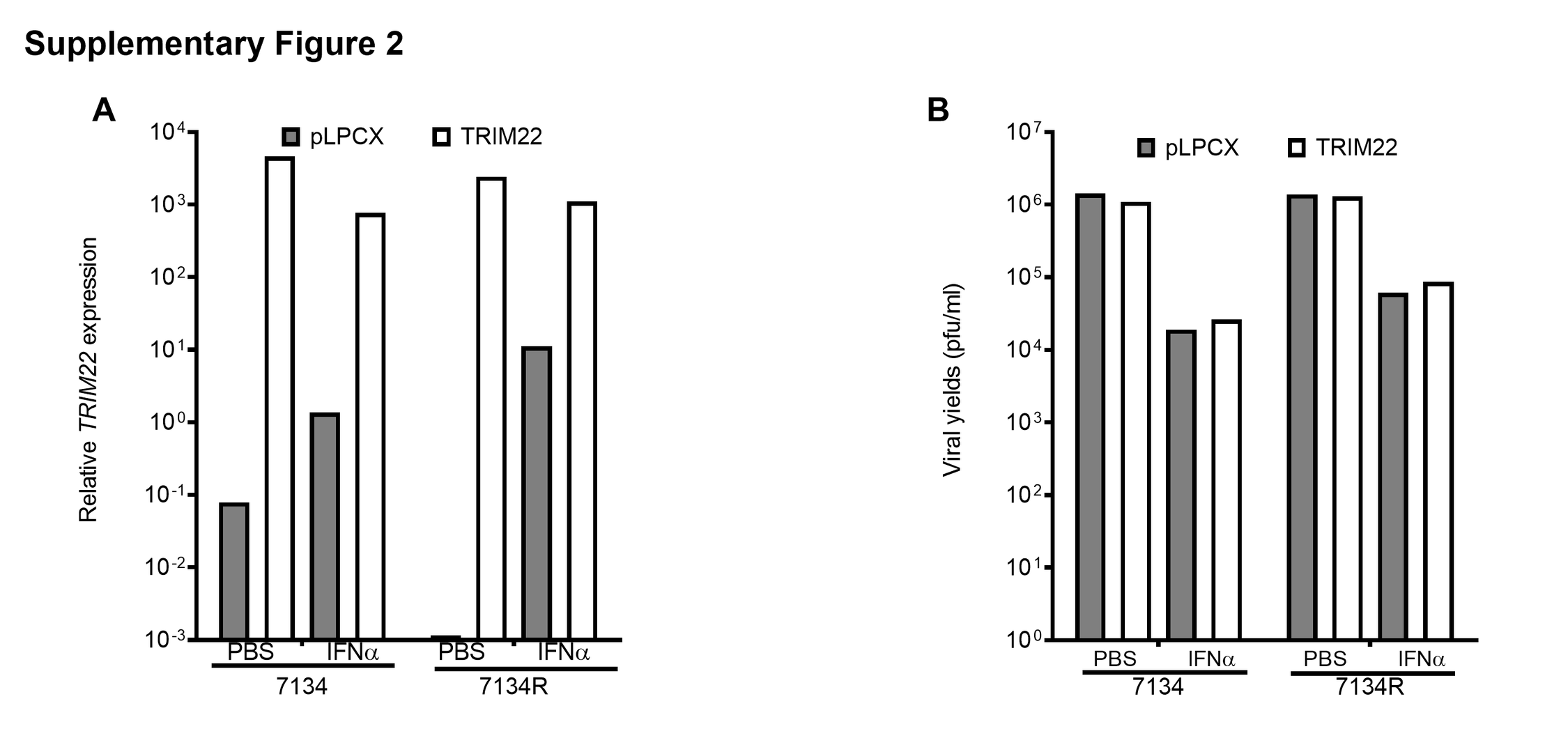

Supplement: S2 Fig — HeLa cells transfected with an empty vector control (pLPCX) or a vector with a TRIM22 insert (TRIM22) were treated with PBS or hIFNα-2a at 1000U/ml for 24 h. Transfected cells were infected with ICP0-null (7134) or rescued virus (7134R) at an MOI of 5. (A) Total cell-associated RNA was harvested at 3 hpi and prepared for qRT-PCR. TRIM22 transcripts were normalized to 18S rRNA. (B) Virus yields at 24 hpi were determined with plaque assays on U2OS cells (n = 1). (TIF) [file ppat.1009281.s002.tif]

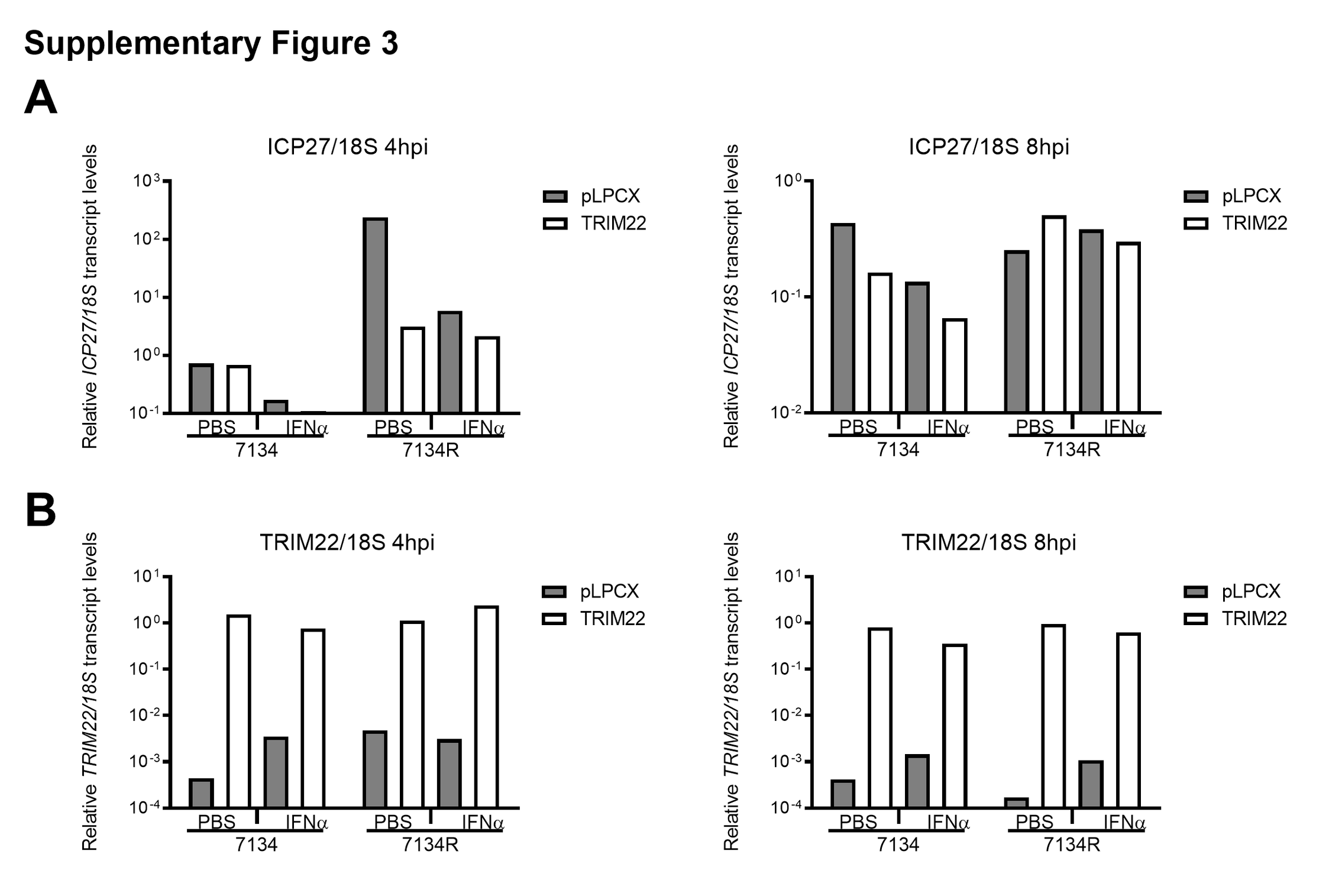

Supplement: S3 Fig — HeLa cells transfected with an empty vector control (pLPCX) or a vector with a TRIM22 insert (TRIM22) were treated with PBS or hIFNα-2a at 1000U/ml for 24 h. Transfected cells were infected with ICP0-null (7134) or rescued virus (7134R) at an MOI of 0.1. (A) Total cell-associated RNA was harvested at 4 hpi and 8hpi and prepared for qRT-PCR. ICP27 transcripts were normalized to 18S at 4 hpi (A) and at 8 hpi (B). TRIM22 transcripts were normalized to 18S rRNA at 4 hpi (C) and at 8 hpi (D) (n = 1). (TIF) [file ppat.1009281.s003.tif]

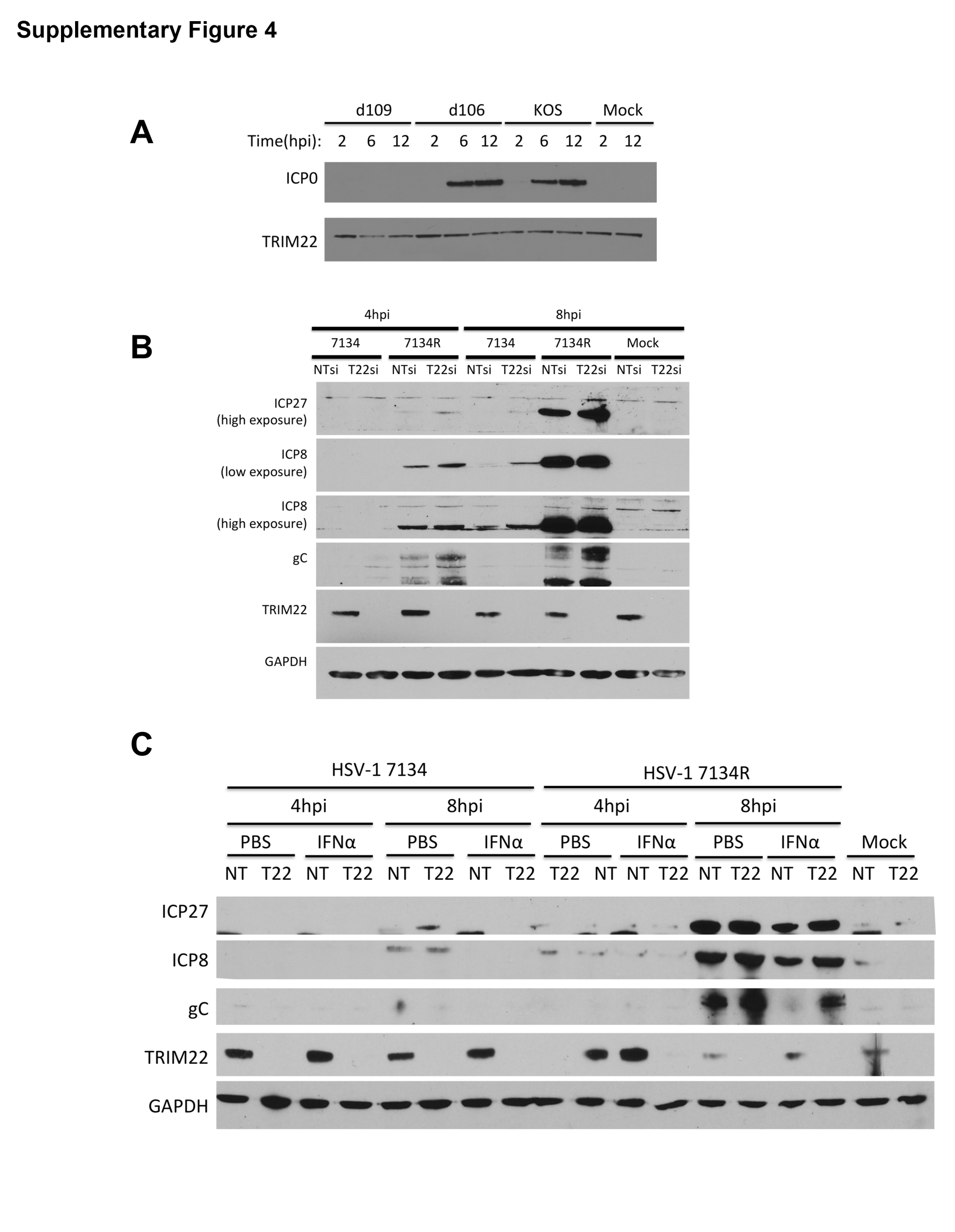

Supplement: S4 Fig — (A) Whole cell lysates from mock-infected or infected with HSV-1 d106, d109, or wildtype KOS viruses (MOI = 5) were probed for ICP0 and TRIM22 at 2 hpi, 6 hpi, and 12 hpi. (B) Whole cell lysates from control-depleted or TRIM22-depleted HFFs that were either mock-infected or infected with 7134 or 7134R viruses (MOI = 5) were probed for TRIM22, GAPDH and the viral proteins ICP27, ICP8, gC, and TRIM22 at 4 hpi and 8 hpi. (C) Whole cell lysates from control-depleted or TRIM22-depleted HFFs that were pre-treated with either PBS or hIFNα (1000U/mL for 24 h) were either mock-infected or infected with 7134 or 7134R viruses (MOI = 5) were probed for TRIM22, GAPDH, and the viral proteins ICP27, ICP8, and gC at 4 hpi and 8 hpi. (TIF) [file ppat.1009281.s004.tif]

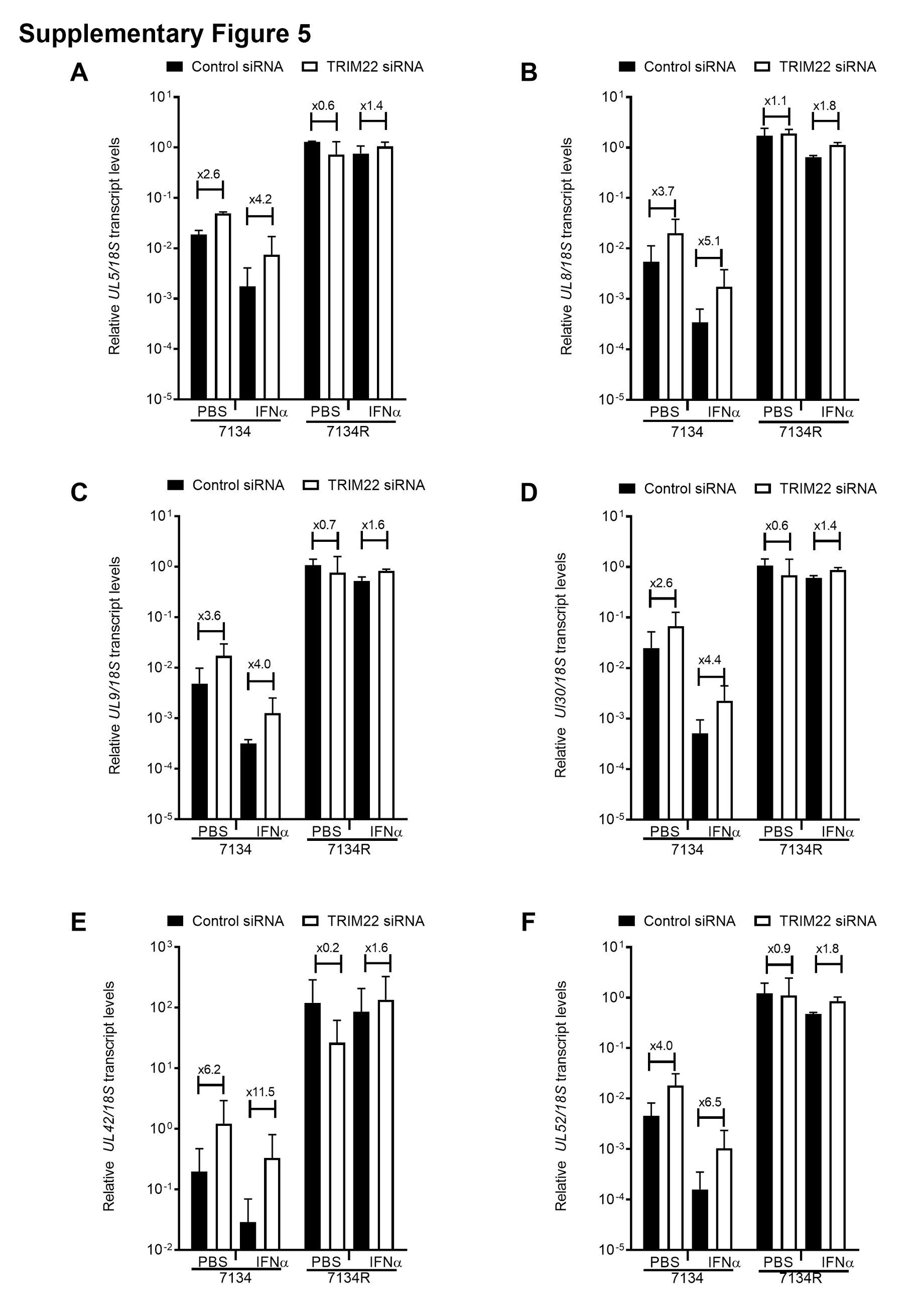

Supplement: S5 Fig — TRIM22-depleted or control-depleted HFFs were treated with PBS or hIFNα-2a at 1000U/ml for 24 h and infected with HSV-1 ICP0-null (7134) or a rescued virus (7134R) at an MOI of 5. Total cell-associated RNA was harvested 8 hpi and transcript levels of UL5 (A), UL8 (B), UL9 (C), UL30 (D), UL42 (E), and UL52 (F) were measured by qRT-PCR. The transcript levels were normalized to 18S rRNA (n = 3). Fold differences due to TRIM22 depletion are shown above corresponding bars. (TIF) [file ppat.1009281.s005.tif]

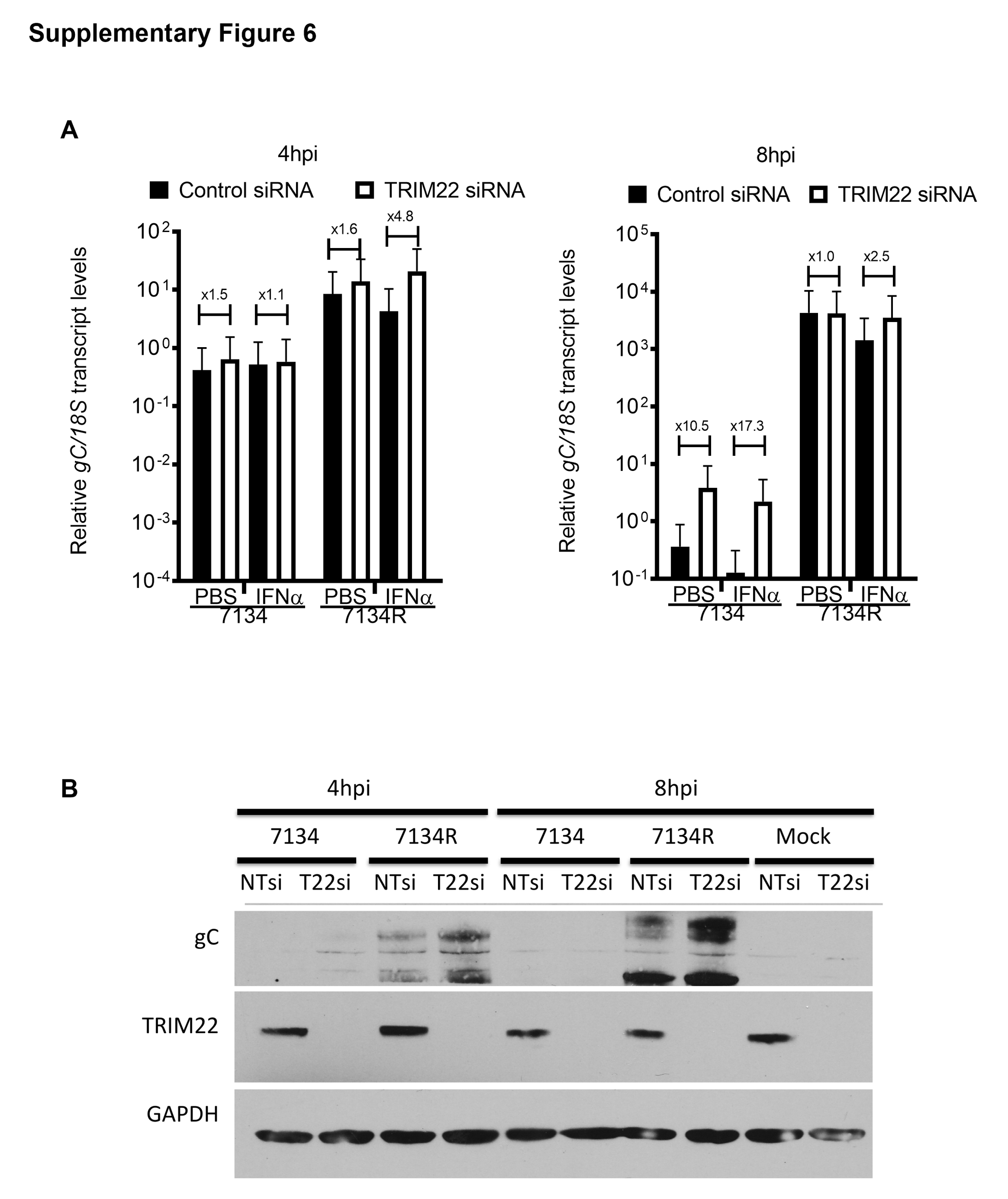

Supplement: S6 Fig — HFFs were transfected with siRNA pools specific for TRIM22 or non-targeting siRNAs and were treated with PBS or hIFNα-2a at 1000U/ml for 24 h and infected with HSV-1 ICP0-null (7134) or a rescued virus (7134R) at an MOI of 5. (A) Total cell-associated RNA was harvested at 4 hpi (left panel) and 8 hpi (right panel) and prepared for qRT-PCR. gC transcripts were measured and normalized to 18S rRNA levels (n = 2) (A). Fold differences due to TRIM22 depletion are shown above corresponding bars. (B) Whole cell lysates were collected at 4 hpi and 8 hpi and the representative western blot shows gC, TRIM22 and GAPDH protein levels. (TIF) [file ppat.1009281.s006.tif]

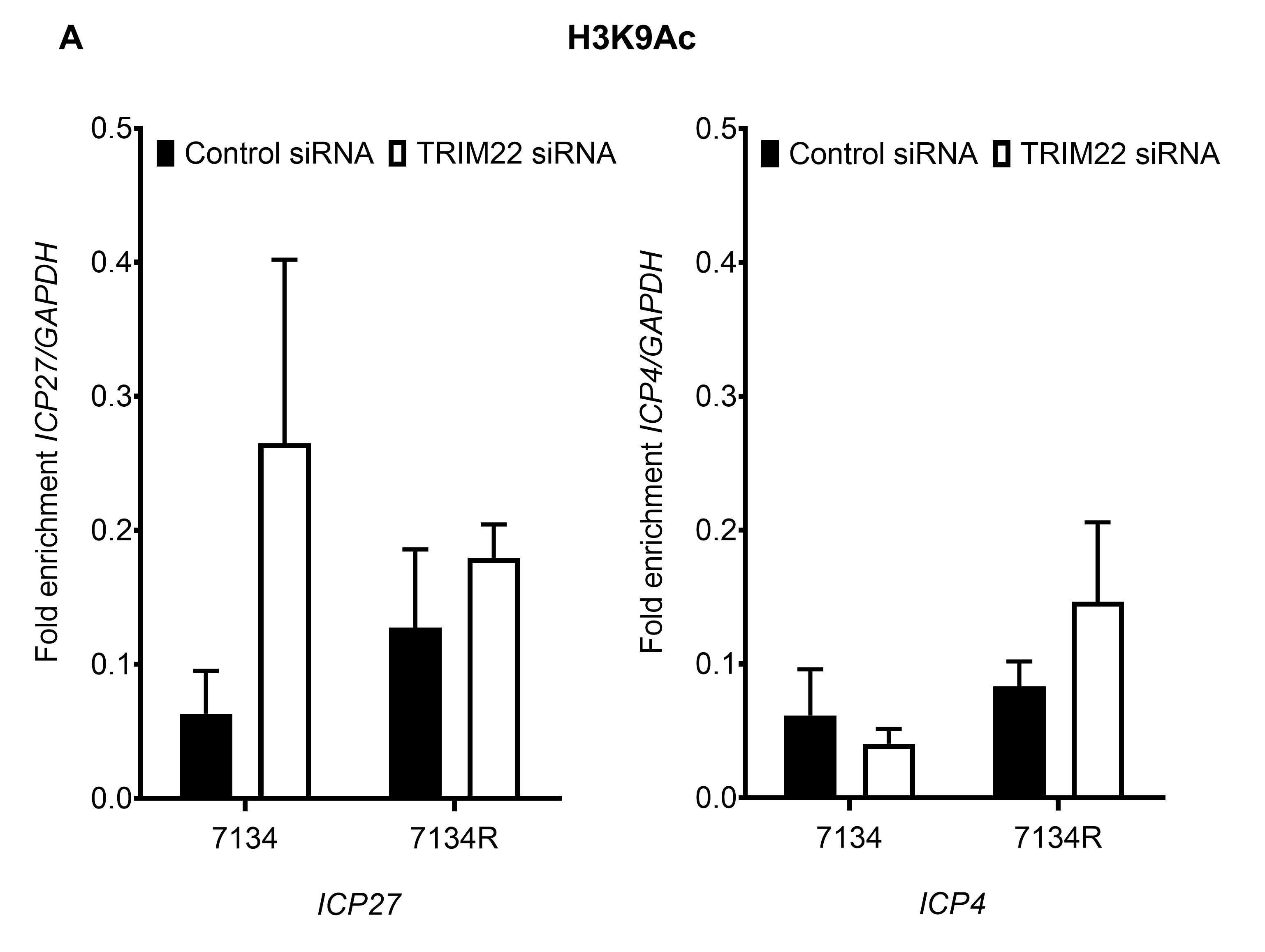

Supplement: S7 Fig — Control- or TRIM22-siRNA transfected HFFs were infected with HSV-1 ICP0-null (7134) or HSV-1 ICP0-rescued (7134R) viruses at an MOI of 5. ChIP was conducted on cell extracts prepared at 6 hpi with antibodies specific for the euchromatin mark H3K9Ac (n = 3) (A). Immunoprecipitated ICP27 (left panel) and ICP4 (right panel) promoter sequences were measured by qPCR and viral DNA sequences were normalized to immunoprecipitated GAPDH DNA. (TIF) [file ppat.1009281.s007.tif]
