# Supplementary material for: Tripartite Motif 22 (TRIM22) protein restricts herpes simplex virus 1 by epigenetic silencing of viral immediate-early genes
Source: PLoS Pathog. 2021 Feb 1;17(2):e1009281. doi: 10.1371/journal.ppat.1009281 (PMC7877759; doi:10.1371/journal.ppat.1009281)
Supplement: S1 Table — (PDF) [file ppat.1009281.s008.pdf]

S1 Table: List of primer sequences used in the study.

Gene	Purpose	Forward primer	Reverse primer
ICP4	qRT-PCR	5′GCGTCGTCGAGGTCGT-3′	5′-CGCGGAGACGGAGGAG-3′
ICP8	qRT-PCR	5’-GGAGGTGCACCGGATACC-3’	5’-GGCTAAAATCCGGCATGAAC-3’
ICP27	qRT-PCR	5′-GCATCCTTCGTGTTTGTCATT-3′	5′-GCATCTTCTCTCC GACCCCG-3′
UL5	qRT-PCR	*5’-AAGACGTTCACCAAGCTGCT-3’*	*5’-CAGATCCACGCCCTTGATGA-3’*
UL8	qRT-PCR	5’ - GGT GAT GAG CGC AGT CC – 3’	5’ - GTC GTG CGT GTC TGT CC – 3’
UL9	qRT-PCR	5’ - CGACGATACCCTGACCCCAAAG – 3’	5’ - CGAAAAGACCCACCTCCTCCG – 3’
UL30	qRT-PCR	*5’-CCCAAGGTGTACTGCGGG-3’*	*5’-CTCCACGTTCTCCAGGATGT-3’*

UL42	qRT-PCR	5’ - ACGTCCGACGGCGAGG – 3’	5’ - CAGGCGCAACTGAACGTC – 3’
UL52	qRT-PCR	5’ - GCCCTCCTCACAAACTCTCTACTG – 3’	5’ - CCGTCCCCAATAAACAAAAGG – 3’
gC	qRT-PCR	5′ - CCCCCGCGGACCTTCACCT - 3′	5′ - GGCCGCGGCAGCACCAG - 3′
h18S RNA	qRT-PCR	5′-GCATTCGTATTGCGCCGCTA-3′	5′-AGCTGCCCGGCGGGT-3′
TRIM22 RNA	qRT-PCR	5′-AGCTCGTTTAGTGAACCGTCAGATC-3′	5′-ACCTACAGGTGGGGTCTTTCATTCCC -3′
ICP8 promoter	qPCR	5′-GCCCGGGCGCTGCTTGTTCTCC-3′	5′-CGTCCGCCGTCGCAGCCGTATC-3′
ICP27 promoter	qPCR	5′-CCGCCGGCCTGGATGTGACG-3′	5′-CGTGGTGGCCGGGGTGGTGCTC-3
ICP4 promoter	qPCR	5′-GCCGTCGACGCGGAACT-3′	5′-CCTTTTTCCCACCCAAGCAT-3′

GAPDH pseudogene	qPCR	5'-CAGGCGCCCAATACGACCAAAATC-3'	5'-TTCGACAGTCAGTCAGCCGCATCTTCTT-3'
