# Supplementary material for: Tripartite Motif 22 (TRIM22) protein restricts herpes simplex virus 1 by epigenetic silencing of viral immediate-early genes
Source: PLoS Pathog. 2021 Feb 1;17(2):e1009281. doi: 10.1371/journal.ppat.1009281 (PMC7877759; doi:10.1371/journal.ppat.1009281)
Supplement: S2 Table — (DOCX) [file ppat.1009281.s009.docx]

**S2 Table: List of antibodies and their dilutions used in the study.**

| Antibody | Purpose | Dilution | Source |
| --- | --- | --- | --- |
| Mouse anti-ICP0 | Western blotting | 1:1000 | EastCoast Bio |
| Mouse anti-ICP4 | Western blotting | 1:2000 | [1] |
| Rabbit anti-ICP8 | Western blotting | 1:5000 | [2] |
| Mouse anti-ICP27 | Western blotting | 1:5000 | EastCoast Bio |
| Rabbit Glycoprotein C | Western blotting | 1:5000 | [3] |
| Rabbit anti-TRIM22 | Western blotting | 1:1000 | Millipore Sigma |
| Mouse anti-GAPDH | Western blotting | 1: 5000 | Applied Biological Materials |
| Mouse anti-histone H1 | Western blotting | 1:1000 | Sigma-Aldrich |
| Mouse Anti-FLAG M2 | Western blotting | 1:1000 | Sigma-Aldrich |
| Anti-mouse secondary | Western blotting | 1: 5000 | Cell Signaling 7076S |
| Anti-rabbit secondary | Western blotting | 1: 5000 | Cell Signaling 7074S |
| Goat anti-mouse Alexa-488 | Indirect immunofluorescence | 1: 500 | Jackson ImmunoResearch |
| Anti-rabbit Alexa 594 | Indirect immunofluorescence | 1:500 | Jackson ImmunoResearch |
| Anti-histone H3, rabbit | Immunoprecipitation | 2.5ug | Abcam ab1791 |
| Anti-H3K9me3, rabbit | Immunoprecipitation | 2.5ug | Abcam ab8898 |
| Anti-histone H3K27me3, rabbit | Immunoprecipitation | 2.5ug | Active Motif 39156 |
| Negative control rabbit IgG | Immunoprecipitation | 2.5ug | NG 1893918 |

**References**

1. Showalter SD, Zweig M, Hampar B. Monoclonal antibodies to herpes simplex virus type 1 proteins, including the immediate-early protein ICP 4. Infect Immun. 1981;34(3):684-92. Epub 1981/12/01. doi: 10.1128/IAI.34.3.684-692.1981. PubMed PMID: 6277788; PubMed Central PMCID: PMCPMC350925.

2. Knipe DM, Senechek D, Rice SA, Smith JL. Stages in the nuclear association of the herpes simplex virus transcriptional activator protein ICP4. J Virol. 1987;61(2):276-84. Epub 1987/02/01. doi: 10.1128/JVI.61.2.276-284.1987. PubMed PMID: 3027360; PubMed Central PMCID: PMCPMC253947.

3. Eisenberg RJ, Ponce de Leon M, Friedman HM, Fries LF, Frank MM, Hastings JC, et al. Complement component C3b binds directly to purified glycoprotein C of herpes simplex virus types 1 and 2. Microb Pathog. 1987;3(6):423-35. Epub 1987/12/01. doi: 10.1016/0882-4010(87)90012-x. PubMed PMID: 2849025.
